# Optimal control of TGF-*β* to prevent formation of pulmonary fibrosis

**DOI:** 10.1371/journal.pone.0279449

**Published:** 2022-12-30

**Authors:** Fateme Bahram Yazdroudi, Alaeddin Malek

**Affiliations:** Department of Applied Mathematics, Faculty of Mathematical Sciences, Tarbiat Modares University, Tehran, Iran; University of Porto Faculty of Engineering: Universidade do Porto Faculdade de Engenharia, PORTUGAL

## Abstract

In this paper, three optimal control problems are proposed to prevent forming lung fibrosis while control is transforming growth factor-*β* (TGF-*β*) in the myofibroblast diffusion process. Two diffusion equations for fibroblast and myofibroblast are mathematically formulated as the system’s dynamic, while different optimal control model problems are proposed to find the optimal TGF-*β*. During solving the first optimal control problem with the regulator objection function, it is understood that the control function gets unexpected negative values. Thus, in the second optimal control problem, for the control function, the non-negative constraint is imposed. This problem is solved successfully using the extended canonical Hamiltonian equations with no flux boundary conditions. Pontryagin’s minimum principle is used to solve the related optimal control problems successfully. In the third optimal control problem, the fibroblast equation is added to a dynamic system consisting of the partial differential equation. The two-dimensional diffusion equations for fibroblast and myofibroblast are transferred to a system of ordinary differential equations using the central finite differences explicit method. Three theorems and two propositions are proved using extended Pontryagin’s minimum principle and the extended Hamiltonian equations. Numerical results are given. We believe that this optimal strategy can help practitioners apply some medication to reduce the TGF-*β* in preventing the formation of pulmonary fibrosis.

## 1 Introduction

Idiopathic pulmonary fibrosis (IPF) describes a condition in which lung tissue becomes thick, stiff, and scarred. The lungs, alveoli, and blood vessels deliver oxygen to the brain, heart, and other organs. As the lung tissue secretes and thickens, oxygen delivery to the lungs becomes more difficult. As a result, organs do not receive the oxygen they need to function correctly [1, 2]. The process of fibrosis formation is similar to the method of scar formation. In both disorders, after cell destruction, macrophages and other cells begin to produce inflammatory mediators (messenger molecules), including transforming growth factor-*β* (TGF-*β*), among the surface called the interstitium, which causes the proliferation and activation of fibroblasts [3, 4]. Alveolar epithelium is injured repeatedly in IPF. The injury causes the loss of alveolar epithelial cells (AECs) [5]. Fibroblasts, identified by alpha-smooth muscle actin (a-SMA) packets, are activated, converted to myofibroblasts, and contracted by them. Myofibroblasts synthesize collagen and other components of the extracellular matrix (ECM), causing connective tissue deposition or accumulation. TGF-*β*, along with AEC-derived essential fibroblast growth factor (bFGF), increase the proliferation of interstitial fibroblasts [6, 7]. Various factors can cause differentiation between fibroblasts and myofibroblasts in IPF, [8–11], including activation of TGF-*β*, platelet-derived growth factor (PDGF), and other inflammatory mediators [12]. A cell called myofibroblast also appears during wound healing, which is similar to both fibroblasts and smooth muscle. The activity of this cell causes the wound to close after injury phenomenon called wound contraction. The essential and effective cells in all fibrotic diseases are myofibroblasts, which are secreted and contracted by signals and mechanically stabilize ECM in the scar tissue [13].

In 2018, Malek and Varjani solved a class of Hamilton Jacobi-bellman equations using pseudospectral methods [14]. In 2019, Malek and Abbasi introduced hyperthermia cancer therapy by domain decomposition methods using strongly continuous semigroups [15]. In 2020, Malek and Abbasi used a pointwise optimal control solution for cancer treatment by hyperthermia with thermal wave bioheat transfer [16]. Khajanchi and Ghosh investigated the combined effects of optimal control in cancer remission [17]. In 2014, Hao presented a mathematical model for sarcoidosis [18]. Inspired by previous findings, Hao et al. proposed a mathematical model for the immune system of interstitial fibrosis. They proved that it might be used to monitor the effectiveness of existing anti-fibrotic drugs or those undergoing clinical trials in non-renal fibrosis [19]. Following that, Hao and his colleagues developed the previous model for the lung organ. They also considered two unique features for pulmonary fibrosis, including M1-derived inflammatory macrophages and M2 anti-inflammatory alveolar macrophages [20]. In 2017, Hao et al. used this model to evaluate the effect of other potential drugs aimed at preventing liver fibrosis [21]. Their model wa has been solved. Our motivation in this article is to present proper mathematical methods to prevent forming lung fibrosis by controlling TGF-*β* in myofibroblast/fibroblast diffusions. For the first-time novel, optimal control problems for myofibroblast and fibroblast equations as two efficient factors in the tissue repair process are proposed. With the knowledge of the authors, up to now, there is no mathematical optimal control problem with partial differential equation (PDE) constraint for preventing the formation of the TGF-*β* in the available literature. Others announces also used for chronic pancreatitis [22].

### 1.1 Motivation, similarities, differences and novelty

**Fig 1:**
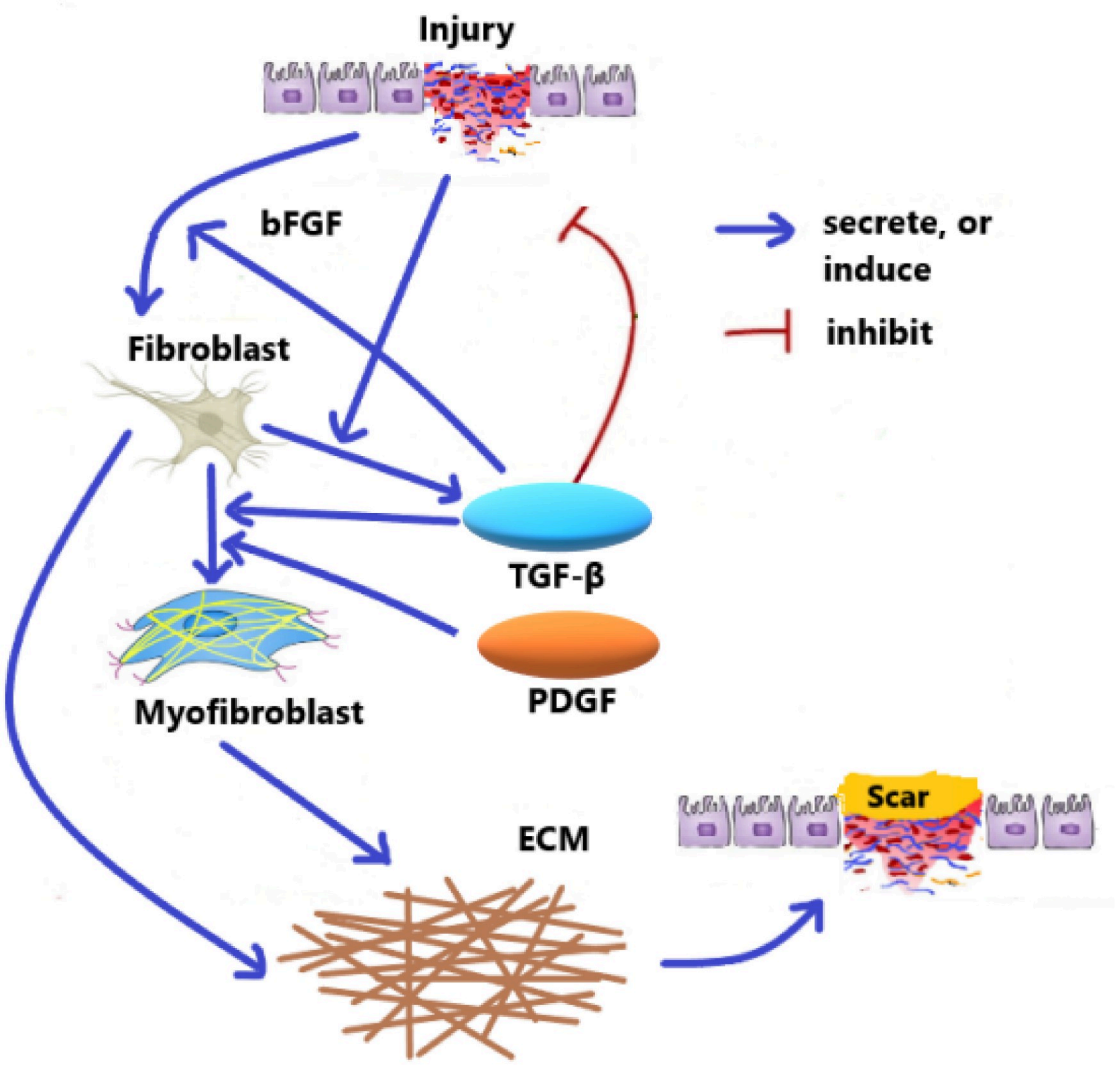
Part of schematic network of cells and proteins in IPF. When the tissue is damaged, the immune system secretes inflammatory mediators such as TGF-*β* and PDGF, which cause myofibroblasts to be active to transfer fibroblasts. Finally, collagen formation takes place in the ECM tissue. The damaged tissue repair. Fibrosis occurs when this repair is excessive.

**Fig 2:**
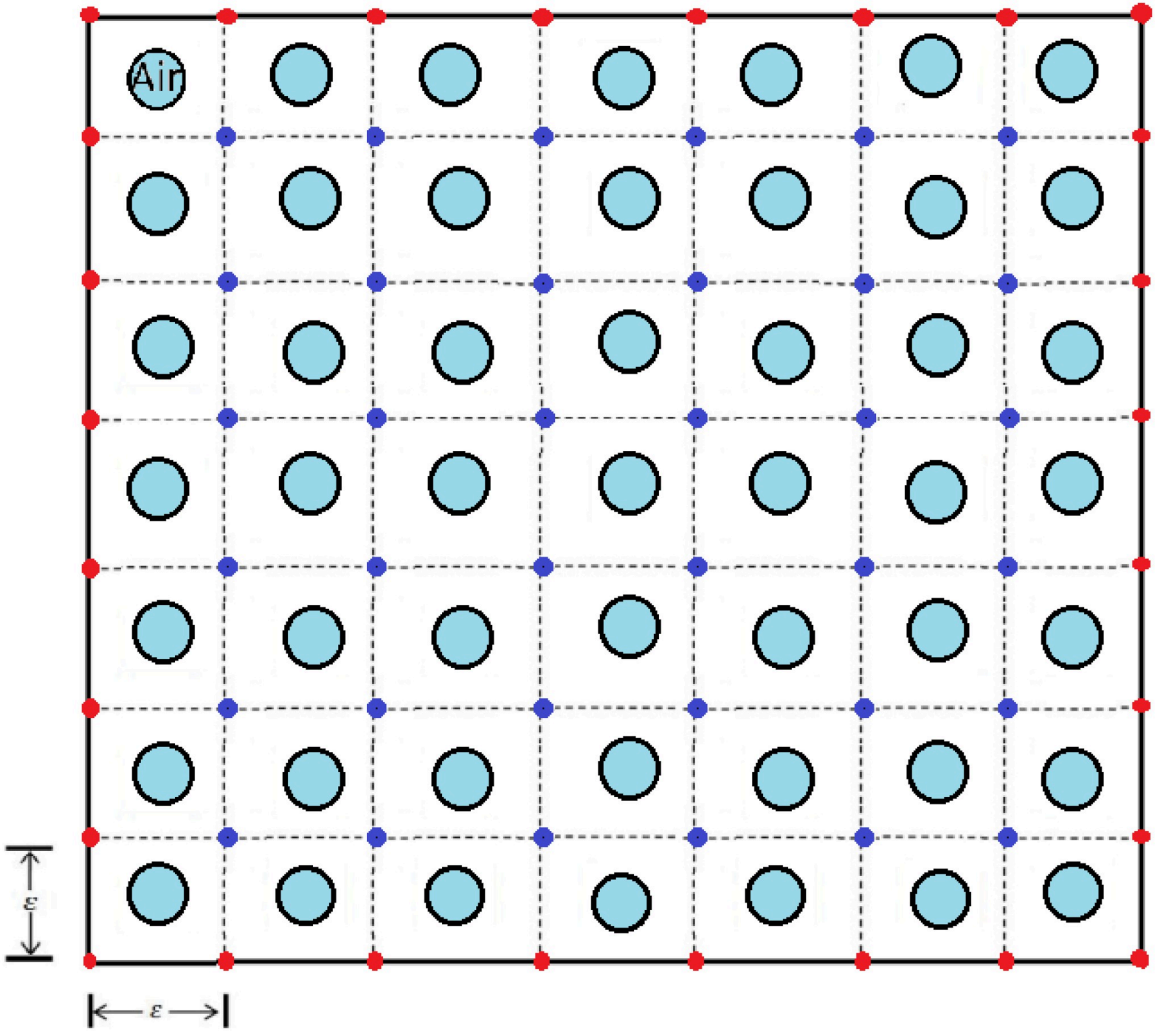
Lung geometry without damage area. Lung geometry consists of squares arranged with smaller circles in the center that show the alveolar air space. Nodes of discretization are indicated by red and blue. The boundary points are shown in red.

**Fig 3:**
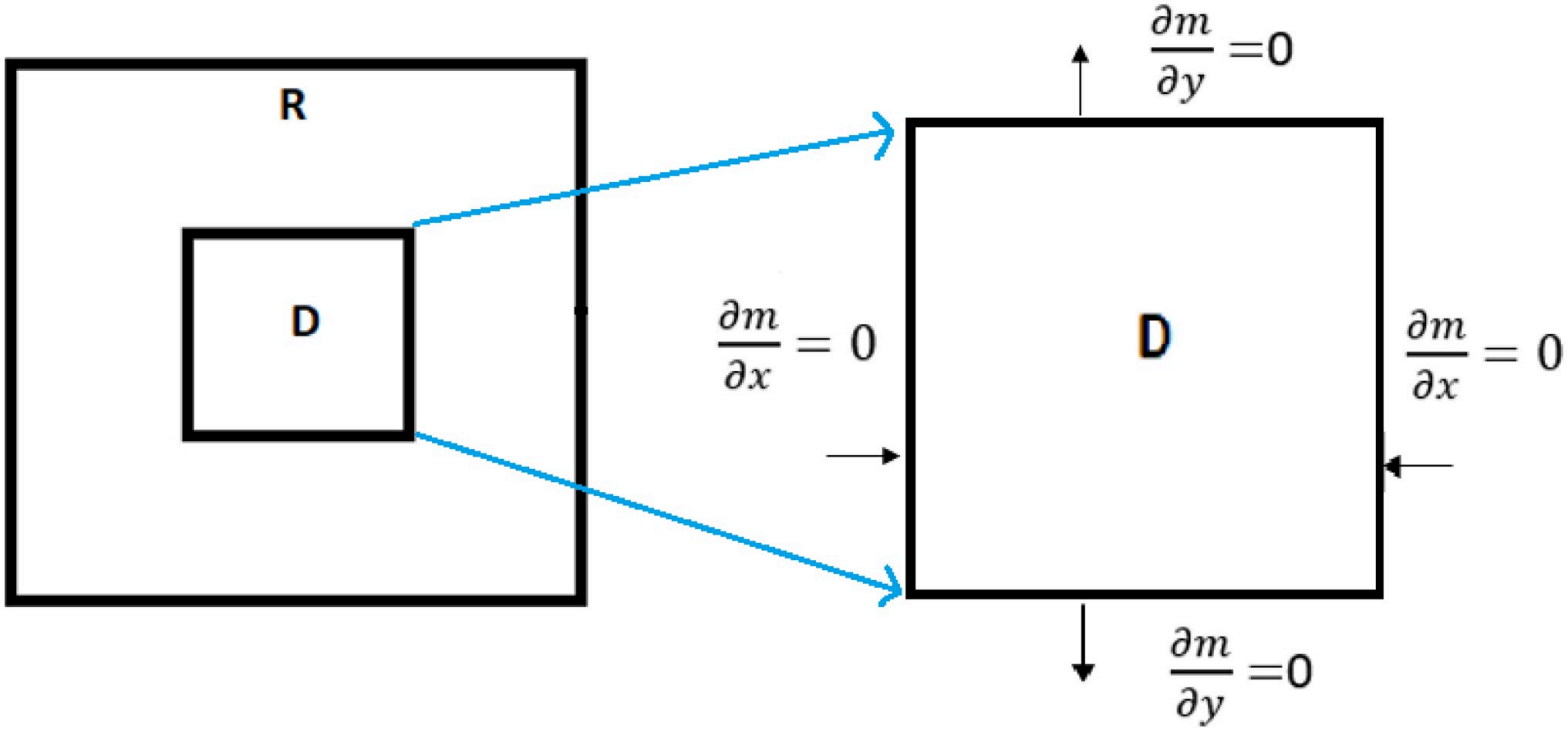
Lung geometry with damage area. The alveolar air space is not considered in the lung geometry. The domain *R* has a damaged area *D*. The boundary conditions have zero flux. We consider the area of inflammation for a mild case of IPF, namely *I*_*D*_ = 0.3 × 0.3 cm^2^ and for a severe case of IPF, namely, *I*_*D*_ = 0.5 × 0.5 cm^2^.

In the presented mathematical models for fibrosis wound [19–21], it is announced that they controlled the relevant dynamic system only by changing the coefficients of parameters in the dynamic system. i.e., no mathematical optimal control problemd a strategy for healing the wound by just changing the coefficient of parameters. Here, in all optimal control problems, we use a dynamical system consisting of PDEs. Moreover, we apply new hybrid methods to solve these types of optimal control problems. We propose five model problems, where three of them are different optimal control problems. Two model problems describe the densities of the fibroblast and myofibroblast. The first model problem is similar to Hao’s model [19]. Other model problems are completely novel. The fibroblast equation in the fourth model problem is different from the fibroblast equation in Hao’s article [19]. The fibroblast equation of us includes terms of transforming fibroblast to myofibroblast by PDGF and transforming fibroblast into ECM. Activated fibroblasts and AECs produce TGF-*β*. We set it in the fibroblast equation. In Section 1, we have an Introduction. In Section 2, IPF is represented schematically by its cells and proteins in Fig 1. As shown in Figs 2 and 3, lung tissue is simulated with and without damage. In the first model problem, we solve a PDE for myofibroblast with the central finite differences explicit method. In the second model problem, we propose an optimal control with the myofibroblast diffusion equation as a dynamic system. We solve the second model problem with the linear-quadratic regulator method yields an infeasible solution. Thus, in Section 3, one constraint that forces the TGF-*β* control variable to stay greater than or equal to the initial value is added to the optimal control as the third model problem. In the third model problem, the extended Hamiltonian method is solved successfully. The reason that we need to solve an extended Hamiltonian equation is:

(i) We face an extra specific ordinary differential equation plus a Riccati differential equation when we write down the liner state feedback low for the control function.
(ii) A constant vector ***b*** appears in the related state space ordinary differential equation (see Eq (15)).

In Section 4, we write the fibroblast diffusion equation as the fourth model problem. Then we solve the optimal control problem with a dynamic system, including the myofibroblast and the fibroblast equations as the fifth model problem. In all five model problems, we convert the diffusion equations into an algebraic system of equations using the central finite differences explicit method. Numerical results are given for the optimal control of TGF-*β*, the myofibroblast density, and the fibroblast density. Section 5 concludes the manuscript. Using the fifth model problem, potential drugs can be tested for their efficacy in stopping the growth of fibrosis in patients.

## 2 Lung tissue simulation

When an injury or infection occurs in an organ, the immune system secretes pro-inflammatory cytokines to suppress and respond to the infection. Inflammatory responses, if excessive, damage the inflamed tissue seriously. Fibrosis is the result of a tissue repair response [23]. According to the referred clinical information in the introduction, the following schematic diagram Fig 1 shows the port of the cellular network and the protein for IPF. For all of the mathematical model problems, we use a simple representation of the lung geometry with two-dimensional. We assume the lung tissue is a square with an edge size of 1 cm. This square is divided by small squares and is called *T*_*ϵ*_ with edge-size *ϵ*. In each small square, there is a concentric circle. It represents the alveolar air space and is called *A*_*ϵ*_. The space between the squares and circles represents the alveolar tissue, as is depicted in Fig 2. We first write down PDE for the alveolar tissue (*T*_*ϵ*_/*A*_*ϵ*_), and then we consider *ϵ* very small and close to zero then obtain the homogenized system. We ignore the alveoli space in the square and call the new space square *R*. In this case, we consider lung tissue to be just a square without alveolar space, as in Fig 3. Tissue inflammation occurs in a small square *D* in *R* (*R* = [0, 1] × [0, 1]).

### 2.1 First model problem (myofibroblast PDE)

For the first model problem, two-dimensional the myofibroblast diffusion equation is as follows [20], where the parameters are given in Table 1 and $\nabla^{2}=\sum \frac{\partial^{2}}{\partial x_{i}\partial y_{j}}$

$$\begin{array}{c} \begin{array}{cc} \frac{\partial m(x,y,t)}{\partial t}-D_{m}\nabla^{2}m\left(x,y,t\right) & =F_{m}\left(m\left(x,y,t\right)\right), \end{array} \end{array}$$ (1)

where

$$\begin{array}{c} \begin{array}{cc} F_{m}\left(m\left(x,y,t\right)\right) & =\underset{f\to m}{\underset{︸}{\left(\lambda_{mfT}\frac{T_{GF}\left(x_{0},y_{0},t_{0}\right)}{K_{T_{GF}}+T_{GF}\left(x_{0},y_{0},t_{0}\right)}+\lambda_{mfG}\frac{G(x_{0},y_{0},t_{0})}{K_{G}+G\left(x_{0},y_{0},t_{0}\right)}\right)}}\\ & \times f\left(x_{0},y_{0},t_{0}\right)-\underset{apoptosis}{\underset{︸}{d_{m}m\left(x,y,t\right)}}. \end{array} \end{array}$$ (2)

**Table 1:** Parameters’ description.

Description		Value
*m*	density of myofibroblasts	*m*(*x*_0_, *y*_0_, *t*_0_) = 8.5 × 10^−3^gcm^−3^ [19, 20]
*f*	density of fibroblasts	*f*(*x*_0_, *y*_0_, *t*_0_) = 4.75 × 10^−3^gcm^−3^ [19, 20]
*G*	concentration of activated PDGF	*G*(*x*_0_, *y*_0_, *t*_0_) = 0.58 × 10^−3^ gcm^−3^ [19, 20]
*T*_*GF*_	concentration of activated TGF-*β*	*T*_*GF*_(*x*_0_, *y*_0_, *t*_0_) = 2.51 × 10^−12^gcm^−3^ [19, 20]
*d*_*m*_	death rate of myofibroblasts	1.66 × 10^−2^day^−1^ [19, 20]
*D*_*m*_	the diffusion coefficient of myofibroblasts	1.47 × 10^−5^cm^2^*day*^−1^ [19, 20]
λ_*mfT*_	activation rate of myofibroblasts due to TGF-*β*	1.2 × 10^−1^day^−1^ [19, 20]
λ_*mfG*_	rate of myofibroblast due to PDGF	1.2 × 10^−1^day^−1^ [19, 20]
*K*_*G*_	PDGF saturation for activation of myofibroblast	1.5 × 10^−8^g/cm^−3^ [19, 20]
$K_{T_{GF}}$	TGF-*β* saturation for alveolar tissue apoptosis	1 × 10^−10^g/cm^−3^ [19, 20]

For two-dimensional discretization, we choose two positive integers *n*_*x*_ = *n*_*y*_ = *n*, *m*(*x*_*i*_, *y*_*j*_, *t*) = *m*_*i*,*j*_(*t*) and 0 ≤ *i* ≤ *n*_*x*_, 0 ≤ *j* ≤ *n*_*y*_, *x*_*i*_ = *i*/*n*_*x*_, *y*_*j*_ = *j*/*n*_*y*_. The central finite differences explicit method of Eq (1) is

$$\begin{array}{c} \begin{array}{cc} \frac{dm_{i,j}\left(t\right)}{\partial t} & =D_{m}(k_{1}^{2}\left[m_{i+1,j}\left(t\right)+m_{i-1,j}\left(t\right)-2m_{i,j}\left(t\right)\right]+k_{2}^{2}[m_{i,j+1}\left(t\right)+m_{i,j-1}\left(t\right)\\ & -2m_{i,j}\left(t\right)])+F\left(m_{i,j}\left(t\right)\right). \end{array} \end{array}$$ (3)

From the known one, we use the following notions *m*(*x*, *y*, *t*) = *m*(*t*), *T*_*GF*_(*x*, *y*, *t*) = *T*_*GF*_(*t*) and *f*(*x*, *y*, *t*) = *f*(*t*).

#### 2.1.1 The homogenized diffusion equation

According to Jikov et al. [24] and Goel et al. [25], the homogenized diffusion equation yields

$$\begin{array}{c} \gamma \frac{\partial m(t)}{\partial t}-D_{m}{\tilde{\nabla }}^{2}m\left(t\right)=\gamma F_{m}\left(m\left(t\right)\right)\;\;\text{in}\;R, \end{array}$$ (4)

where $\gamma =\frac{127}{343}$, ${\tilde{\nabla }}^{2}=\sum a_{ij}\frac{\partial^{2}}{\partial x_{i}\partial y_{j}}$ and *a*_*ii*_ = 0.11 (for *i* = 1, 2). For

$$\begin{array}{c} a=\{\begin{array}{c} a_{ij}=0.11\;\;i=j,\\ a_{ij}=0\;\;\;i\neq j, \end{array} \end{array}$$ (5)

and from (4) and (5)

$$\begin{array}{c} \frac{\partial m(t)}{\partial t}-\frac{a}{\gamma }D_{m}\nabla^{2}m\left(t\right)=F_{m}\left(m\left(t\right)\right)\;\;\text{in}\;R. \end{array}$$ (6)

#### 2.1.2 Second model problem

For the second model problem, we propose an optimal control problem for the myofibroblast diffusion equation using the following initial and boundary conditions.

$$\begin{array}{c} \begin{array}{cc} \underset{m,T_{GF}}{\text{min}}J\left(m,T_{GF},t\right) & =\frac{1}{2}\int_{t_{0}}^{t_{f}}(m{\left(t\right)}^{T}m\left(t\right)+{\left(\frac{T_{GF}\left(t\right)}{K_{T_{GF}}+T_{GF}\left(t\right)}\right)}^{T}\frac{T_{GF}\left(t\right)}{K_{T_{GF}}+T_{GF}\left(t\right)})dt,\\ & s.t. \end{array} \end{array}$$ (7)

$$\begin{array}{c} \begin{array}{cc} \frac{\partial m(t)}{\partial t}-\frac{a}{\gamma }D_{m}\nabla^{2}m\left(t\right) & =(\lambda_{mfT}\frac{T_{GF}\left(t\right)}{K_{T_{GF}}+T_{GF}\left(t\right)}+\lambda_{mfG}\frac{G(t_{0})}{K_{G}+G\left(t_{0}\right)})f\left(t_{0}\right)\\ & -d_{m}m\left(t\right), \end{array} \end{array}$$ (8)

$$\begin{array}{c} \begin{array}{cc} & m\left(t_{0}\right)=8.5\times 10^{-3},\;\text{initial}\;\text{condition}\;\text{at}\;t_{0},\\ & \frac{\partial m(t)}{\partial x}=0,\;\text{boundary}\;\text{conditions}\;\text{for}\;x=0,1,\\ & \frac{\partial m(t)}{\partial y}=0,\;\text{boundary}\;\text{conditions}\;\text{for}\;y=0,1. \end{array} \end{array}$$ (9)

We set

$$\begin{array}{c} U\left(t\right)=\frac{T_{GF}\left(t\right)}{K_{T_{GF}}+T_{GF}\left(t\right)}, \end{array}$$ (10)

thus

$$\begin{array}{c} T_{GF}\left(t\right)=\frac{K_{T_{GF}}U\left(t\right)}{1-U(t)}. \end{array}$$ (11)

We use (10) in (7) and (8) and we have

$$\begin{array}{c} \begin{array}{cc} \underset{m,U_{GF}}{\text{min}}J\left(m,U_{GF},t\right) & =\frac{1}{2}\int_{t_{0}}^{t_{f}}(m{\left(t\right)}^{T}m\left(t\right)+U^{T}\left(t\right)U\left(t\right))dt,\\ & s.t. \end{array} \end{array}$$ (12)

$$\begin{array}{c} \begin{array}{cc} \frac{\partial m(t)}{\partial t}-\frac{a}{\gamma }D_{m}\nabla^{2}m\left(t\right) & =(\lambda_{mfT}U\left(t\right)+\lambda_{mfG}\frac{G(t_{0})}{K_{G}+G\left(t_{0}\right)})f\left(t_{0}\right)-d_{m}m\left(t\right), \end{array} \end{array}$$ (13)

$$\begin{array}{c} \begin{array}{cc} & m\left(t_{0}\right)=8.5\times 10^{-3},\;\;\text{initial}\;\text{condition}\;\text{at}\;t_{0},\\ & \frac{\partial m(t)}{\partial x}=0,\;\text{boundary}\;\text{conditions}\;\text{for}\;x=0,1,\\ & \frac{\partial m(t)}{\partial y}=0,\;\text{boundary}\;\text{conditions}\;\text{for}\;y=0,1. \end{array} \end{array}$$ (14)

The linear-quadratic regulator is efficient in the optimal control problems [26]. We use the linear-quadratic regulator for solving, but Eq (13) is non-linear. For this purpose, we first convert Eq (13) to a linear form. For discretization, we use the second-order central finite differences. Using the extension of Smith’s work [27] for the one-dimensional problem to the system (see also [28]), the block form of Eq (13) is as follows:

$$\begin{array}{c} \frac{dm}{dt}=Am\left(t\right)+BU\left(t\right)+b. \end{array}$$ (15)

We have discrete the linear equation system, where block matrices of Eq (15) are as follows

$$\begin{array}{cc} A & ={\left[\begin{array}{cccccc} G_{m} & L^{T} & O & O & \cdots & O\\ L & G_{m} & L^{T} & O & \cdots & O\\ O & L & G_{m} & L^{T} & \cdots & O\\ \vdots & \; & \ddots & \ddots & \ddots & \vdots \\ O & O & \cdots & O & L & G_{m} \end{array}\right]}_{{\left(n-1\right)}^{2}\times {\left(n-1\right)}^{2}}, \end{array}$$

$$\begin{array}{cc} G_{m} & ={\left[\begin{array}{ccccc} -2r_{m}-d_{m} & r_{m} & 0 & \cdots & 0\\ r_{m} & -2r_{m}-d_{m} & r_{m} & \cdots & 0\\ 0 & r_{m} & -2r_{m}-d_{m} & \cdots & 0\\ \vdots & \ddots & \ddots & \ddots & r_{m}\\ 0 & \cdots & 0 & r_{m} & -2r_{m}-d_{m} \end{array}\right]}_{\left(n-1)\times (n-1\right)}, \end{array}$$

$$\begin{array}{cc} L & ={\left[\begin{array}{ccccc} 0 & 0 & 0 & \cdots & 0\\ r_{m} & 0 & 0 & \cdots & 0\\ 0 & r_{m} & 0 & \cdots & 0\\ \vdots & \ddots & \ddots & \ddots & \vdots \\ 0 & \cdots & 0 & r_{m} & 0 \end{array}\right]}_{\left(n-1)\times (n-1\right)}, \end{array}$$

in which, $r_{m}=\frac{a(D_{m}\times dx^{2})}{\gamma }$. Moreover

$$\begin{array}{c} B={\left[\begin{array}{ccccc} \theta_{m}I & I & 0 & \cdots & 0\\ I & \theta_{m}I & I & \cdots & 0\\ 0 & I & \theta_{m}I & \cdots & 0\\ \vdots & \ddots & \ddots & \ddots & I\\ 0 & \cdots & 0 & I & \theta_{m}I \end{array}\right]}_{{\left(n-1\right)}^{2}\times {\left(n-1\right)}^{2}}, \end{array}$$

in which, *θ*_*m*_ = λ_*mft*_*f*(*t*_0_)

$$\begin{array}{c} b={\left[\begin{array}{c} b_{1}\\ b_{2}\\ \vdots \\ b_{n} \end{array}\right]}_{{\left(n-1\right)}^{2}\times 1}b_{j}=r{\left[\begin{array}{c} m_{1,j}\left(t\right)\\ 0\\ \vdots \\ 0\\ m_{n,j}\left(t\right) \end{array}\right]}_{(n-1)\times 1}+c_{m}{\left[\begin{array}{c} 1\\ 1\\ \vdots \\ 1\\ 1 \end{array}\right]}_{(n-1)\times 1}, \end{array}$$

in which, $c_{m}=\left(\lambda_{mfG}\frac{G(t_{0})}{K_{G}+G\left(t_{0}\right)}\right)f\left(t_{0}\right)$, *m*_*i*,*j*_(*t*_0_) = 8.5 × 10^−3^ and

$$\begin{array}{c} \begin{array}{c} m\left(t\right)={\left[\begin{array}{c} m_{1}\left(t\right)\\ m_{2}\left(t\right)\\ \vdots \\ m_{n}\left(t\right) \end{array}\right]}_{{\left(n-1\right)}^{2}\times 1}m_{j}\left(t\right)={\left[\begin{array}{c} m_{1,j}\left(t\right)\\ m_{2,j}\left(t\right)\\ \vdots \\ m_{n,j}\left(t\right) \end{array}\right]}_{(n-1)\times 1}. \end{array} \end{array}$$ (16)

*I* is identity matrix, ***b***_***j***_ is a column vector of zeros and known boundary values which are added with a fixed value of *c*_*m*_.

#### 2.1.3 Solution of the linear-quadratic regulator

The minimization of the performance index *J* will be done using Pontryagin’s minimum principle [14, 29]. The Hamiltonian is

$$\begin{array}{c} \begin{array}{cc} H(m(t),U(t),\lambda (t),t) & =m{\left(t\right)}^{T}m\left(t\right)+U{\left(t\right)}^{T}U\left(t\right)\\ & +\lambda^{T}\left(t\right)(Am(t)+BU(t)+b), \end{array} \end{array}$$ (17)

where **λ** is the vector of Lagrange multipliers. Define $\hat{J}$ is as follows

$$\begin{array}{c} \begin{array}{cc} \hat{J} & =\frac{1}{2}\int_{t_{0}}^{t_{f}}(m{\left(t\right)}^{T}m\left(t\right)+U{\left(t\right)}^{T}U\left(t\right)+\lambda^{T}\left(t\right)\left[Am\left(t\right)+BU\left(t\right)+b-\dot{m}\left(t\right)\right])dt. \end{array} \end{array}$$ (18)

By substitute Eq (17) in Eq (18), we have

$$\begin{array}{c} \begin{array}{cc} \hat{J} & =\int_{t_{0}}^{t_{f}}\left[H\left(m\left(t\right),U\left(t\right),\lambda \left(t\right),t\right)-\lambda^{T}\left(t\right)\dot{m}\left(t\right)\right]dt. \end{array} \end{array}$$ (19)

We apply the integration in Eq (19), we have

$$\begin{array}{c} \begin{array}{cc} \hat{J} & =-\left[\lambda^{T}\left(t\right)m\left(t\right)\right]{|}_{t_{0}}^{t_{f}}+\int_{t_{0}}^{t_{f}}\left[H\left(m\left(t\right),U\left(t\right),\lambda \left(t\right),t\right)-{\dot{\lambda }}^{T}\left(t\right)m\left(t\right)\right]dt. \end{array} \end{array}$$ (20)

The first variation $\delta \hat{J}$ with respect to the vectors ***m*** and ***U*** is given by

$$\begin{array}{c} \begin{array}{c} -\lambda^{T}\left(t_{f}\right)+\int_{t_{0}}^{t_{f}}[\delta m\left(t\right)\left[\frac{\partial H}{\partial m}+\dot{\lambda }\right]+\delta U^{T}\frac{\partial H}{\partial U}]dt. \end{array} \end{array}$$ (21)

The necessary condition for $\hat{J}$ to have a minimum or a maximum is that $\delta \hat{J}=0$, for every *δ* ***m*** and *δ* ***U***_***G*** ***F***_, the vectors ***m*** and ***U***_***G*** ***F***_ must satisfy in the follows equations

$$\begin{array}{c} \left\{ \begin{array}{c} \frac{\partial H(m^{*}\left(t\right),U^{*}\left(t\right),\lambda^{*}\left(t\right),t))}{\partial m}=-{\dot{\lambda }}^{*}\left(t\right)\;i.e.\;{\dot{\lambda }}^{*}\left(t\right)=-m^{*}\left(t\right)-A^{T}\lambda^{*}\left(t\right),\\ \frac{\partial H(m^{*}\left(t\right),U^{*}\left(t\right),\lambda^{*}\left(t\right),t)}{\partial U}=0\;i.e.\;U^{*}\left(t\right)+B^{T}\lambda^{*}\left(t\right)=0,\\ \frac{\partial H(m^{*}\left(t\right),U^{*}\left(t\right),\lambda^{*}\left(t\right),t)}{\partial \lambda }={\dot{m}}^{*}\;i.e.\;{\dot{m}}^{*}=Am^{*}\left(t\right)+BU^{*}\left(t\right)+b. \end{array}\right. \end{array}$$ (22)

In this case, we have that

$$\begin{array}{c} U^{*}\left(t\right)=-B^{T}\lambda^{*}\left(t\right). \end{array}$$ (23)

A way to find the optimal control is linear feedback form; that is, to look for function *K*(*t*) and ***ρ***(*t*) for which

$$\begin{array}{c} \begin{array}{c} U^{*}\left(t\right)=K\left(t\right)m^{*}\left(t\right)+\rho \left(t\right). \end{array} \end{array}$$ (24)

For the unknowns ***ρ***(*t*) and *K*(*t*) as the feedback metrics. We assume that the vector of Lagrange multipliers λ*(*t*) is linear in m*(*t*), i.e.

$$\begin{array}{c} \lambda^{*}\left(t\right)=p\left(t\right)m^{*}\left(t\right)-b\eta \left(t\right), \end{array}$$ (25)

for the unknowns ***p***(*t*) and ***η***(*t*), if we substitute Eq (25) in Eq (23), we have

$$\begin{array}{c} \begin{array}{c} U^{*}\left(t\right)=-B^{T}p\left(t\right)m^{*}\left(t\right)+B^{T}b\eta \left(t\right). \end{array} \end{array}$$ (26)

By comparing (26) with (24), we have

$$\begin{array}{c} K\left(t\right)=-B^{T}p\left(t\right),\;\rho \left(t\right)=B^{T}b\eta \left(t\right). \end{array}$$

By substitute Eq (26) in Eq (15), we have

$$\begin{array}{c} \begin{array}{c} {\dot{m}}^{*}\left(t\right)=Am^{*}\left(t\right)+B\left(-B^{T}p\left(t\right)m^{*}\left(t\right)+B^{T}b\eta \left(t\right)\right)+b. \end{array} \end{array}$$ (27)

From differentiate Eq (25) and use (22), we have

$$\begin{array}{c} \begin{array}{c} {\dot{\lambda }}^{*}\left(t\right)=\dot{p}\left(t\right)m^{*}\left(t\right)+p\left(t\right){\dot{m}}^{*}\left(t\right)-b\dot{\eta }\left(t\right)=-m^{*}\left(t\right)-A^{T}\lambda^{*}\left(t\right). \end{array} \end{array}$$ (28)

Finally, if we substitute Eq (27) in Eq (28) and use Eq (25), we arrive at the relation

$$\begin{array}{c} \begin{array}{cc} m^{*}\left(t\right)(\dot{p}\left(t\right)+p\left(t\right)A+A^{T}p\left(t\right)-p\left(t\right)BB^{T}p\left(t\right)+I) & +\\ b(-\dot{\eta }\left(t\right)-\eta \left(t\right)\left(A-B^{T}p\left(t\right)B\right)+p\left(t\right)) & =0. \end{array} \end{array}$$ (29)

m*(*t*) and ***b*** are not zero. For Eq (29) to be valid, the coefficient of m*(*t*) and the second term in Eq (29) must simultaneously be equal to zero. This reduces Eq (29) to the following two differential equations:

$$\begin{array}{c} \dot{p}\left(t\right)+p\left(t\right)A+A^{T}p\left(t\right)-p\left(t\right)BB^{T}p\left(t\right)=-I, \end{array}$$ (30)

by calculating ***p***(*t*) from (30), one calculat ***η***(*t*) by (31)

$$\begin{array}{c} \dot{\eta }\left(t\right)+\eta \left(t\right)\left(A-B^{T}p\left(t\right)B\right)=p\left(t\right). \end{array}$$ (31)

Thus the following theorem and proposition are held for the second model problem.

*Theorem 2.1.* A minimum ${\hat{J}}^{*}$ exists if and only if the solution ***p***(*t*) of the Riccati equation (30) exist, is bounded, and is positive definite for all *t* < *t*_*f*_. In this case, the minimum performance index ${\hat{J}}^{*}$ becomes

$$\begin{array}{c} {\hat{J}}^{*}=\frac{1}{2}m{*}^{T}\left(t_{0}\right)p\left(t_{0}\right)m^{*}\left(t_{0}\right). \end{array}$$ (32)

**Proof**: See Section 2.1.3. (For the related theorem for example see theorem 11.3.1 page 496, [29]).

*Proposition 2.1.* In practice, the optimal control problem (12)–(14) is solved and the optimal value is calculated by Eq (24). Using Eqs (11) and (24), the second model problem (7)–(9) can be solved and its optimal value is as follows

$$\begin{array}{c} T_{GF}^{*}\left(t\right)=\frac{K_{T_{GF}}\left(K\left(t\right)m^{*}\left(t\right)+\rho \left(t\right)\right)}{1-K\left(t\right)m^{*}\left(t\right)-\rho \left(t\right)}. \end{array}$$ (33)

### 2.2 Numerical results

We study the numerical aspect of the optimal control problem to validate our analytical results in the previous sections. The Algorithm 1 is as follows:

- Step 1: We convert Eq (6) to Eq (15) using the discretization of second-order central finite differences.
- Step 2: We set the initial value fram Table 1 for TGF-*β* control and myofibroblast state.
- Step 3: We solve Eqs (30) and (31) with the Euler approximation to find ***P*** and ***η***.
- Step 4: For obtaining TGF-*β* control, we substitute ***P*** and ***η*** in Eq (26). Then substitute known values in Eq (26) in to Eq (11).
- Step 5: For obtaining myofibroblast state, we substitute TGF-*β* in Eq (15).
- Step 6: We update the control and state in each iteration by using the values of the optimality system obtained in the previous iterations.
- Step 7: The procedure is continued iteratively till the convergence is achieved.

Results are computed for 300 days using python programming software version 3.8 in spider idle. The processor is Intel(R), Core(TM), i5–7500 CPU.

**Fig 4:**
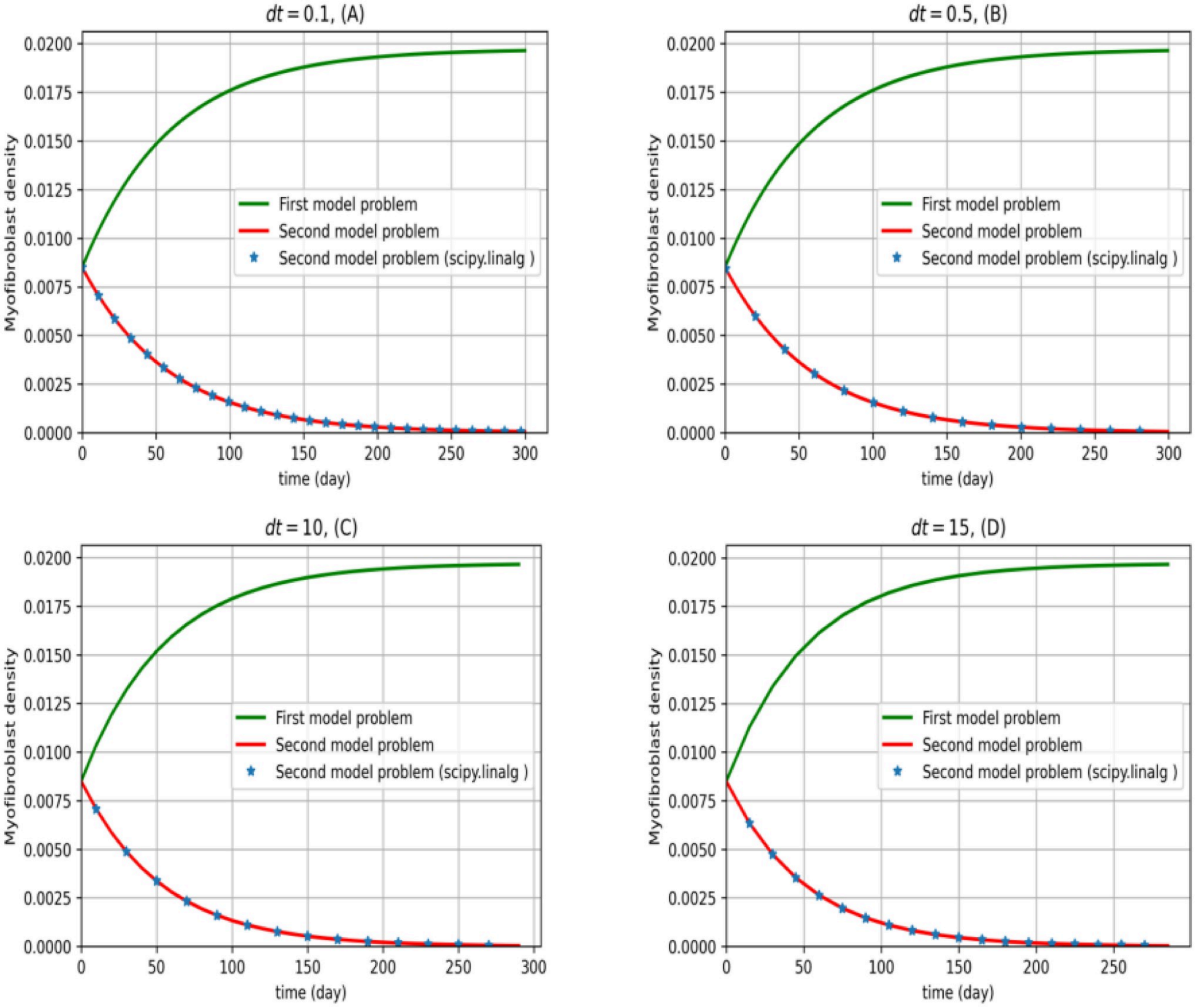
First and second model problems and comparison with a package. Myofibroblast density against time is plotted. The first model problem (dynamical system (8) and (9)) is solved by the central finite differences explicit method (green). The second model problem (7)–(9) by scipy.linalg package (⋆) and current technique (red) are solved. Plots are depicted for *dt* = 0.1, 0.5, 10, 15 in Euler approximation. As it is shown, the myofibroblast vanishes to zero for all the *dt* values. However, from a mathematical point of view, the results for *dt* = 0.1 are more reasonable. This means that after almost 240 days of controlling TGF-*β*, the myofibroblast vanishes. All calculations are done for 36 nodes in the x-y plane.

**Fig 5:**
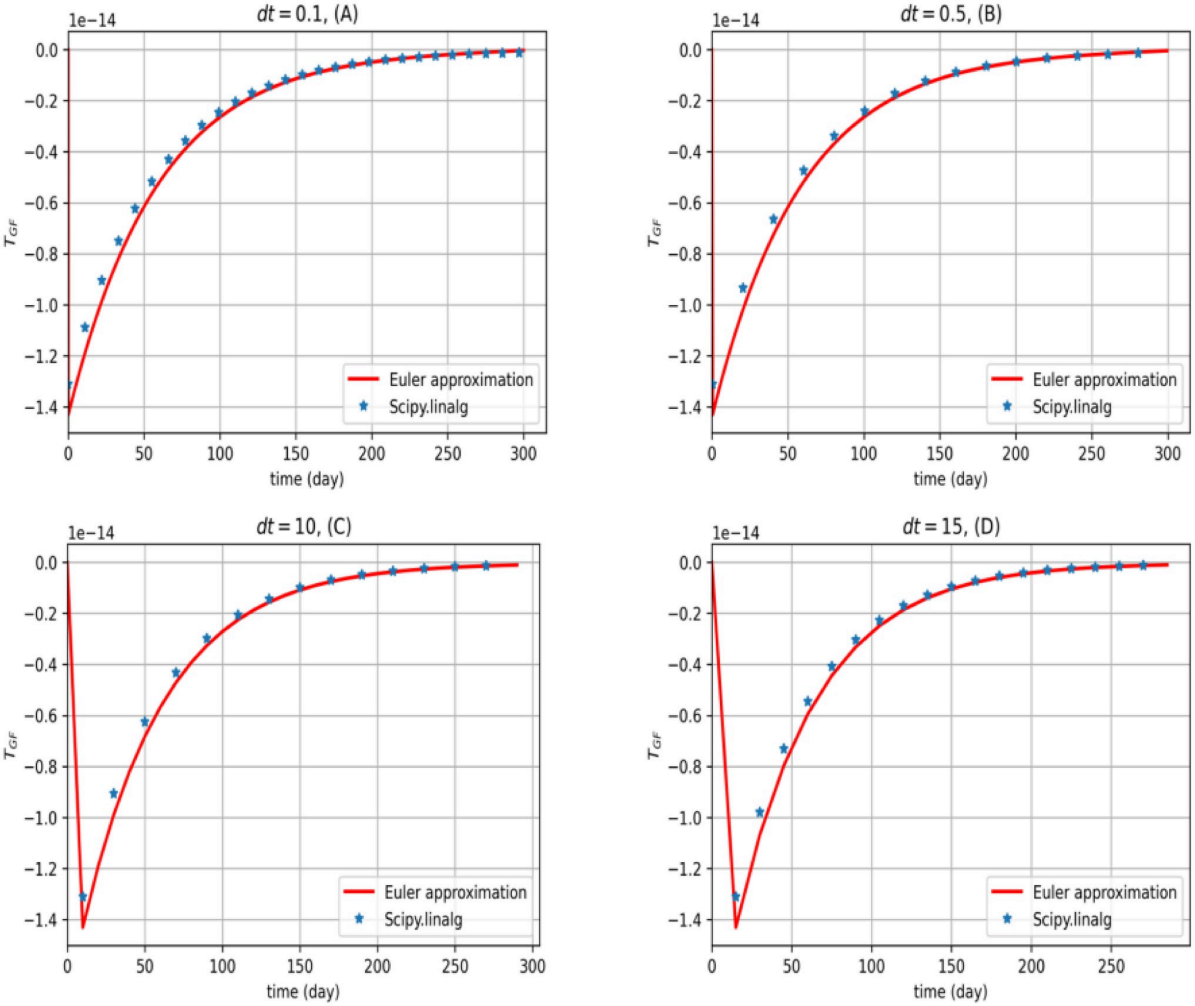
The optimal control function for TGF-β. The second model problem (7)–(9) is solved. Graphs of optimal TGF-*β* are depicted for *dt* = 0.1, 0.5, 10, 15. As is shown, the Euler approximation and the scipy.linalg package has almost the same results. However, since TGF-*β* in the real world can not take negative values, one finds out that the second model problem (7)–(9) must be improved. In solving the central finite differences explicit method, all calculations are done for 36 nodes in the x-y plane.

In the following diagram, we display the effect of treatment for a mild case of IPF, namely *I*_*D*_ = 0.3 × 0.3. The linear-quadratic regulator method is used for the modeling of least myofibroblast diffusion. We repeat the average myofibroblast density and the average TGF-*β* concentration from homeostasis with different periods. The results are shown in Figs 4 and 5. We use a solve-continuous-are function from scipy.linalg package for Riccati equation.

## 3 Third model problem

In Section 2, we do not have any constrained on TGF-***β***. Thus one needs to modify the previous optimal control problem. In this section, it is assumed that TGF-***β*** has a non-negative constraint. We have proposed an optimal control problem as follows.

$$\begin{array}{c} \begin{array}{cc} \underset{m,T_{GF}}{\text{min}}J\left(m\left(t\right),T_{GF}\left(t\right),t\right) & =\frac{1}{2}\int_{t_{0}}^{t_{f}}(m{\left(t\right)}^{T}m\left(t\right)\\ & +{\left(\frac{T_{GF}\left(t\right)}{K_{T_{GF}}+T_{GF}\left(t\right)}\right)}^{T}\frac{T_{GF}\left(t\right)}{K_{T_{GF}}+T_{GF}\left(t\right)})dt,\\ & s.t. \end{array} \end{array}$$ (34)

$$\begin{array}{c} \begin{array}{cc} \frac{\partial m(t)}{\partial t}-\frac{a}{\gamma }D_{m}\nabla^{2}m\left(t\right) & =\lambda_{mfT}(\frac{T_{GF}\left(t\right)}{K_{T_{GF}}+T_{GF}\left(t\right)}+\lambda_{mfG}\frac{G(t_{0})}{K_{G}+G\left(t_{0}\right)})f\left(t_{0}\right)\\ & -d_{m}m\left(t\right), \end{array} \end{array}$$ (35)

$$\begin{array}{c} \begin{array}{c} \frac{K_{T_{GF}}T_{GF}\left(t_{0}\right)}{1-T_{GF}\left(t_{0}\right)}\leq \frac{K_{T_{GF}}T_{GF}\left(t\right)}{1-T_{GF}\left(t\right)}, \end{array} \end{array}$$ (36)

$$\begin{array}{c} \begin{array}{cc} & m\left(t_{0}\right)=8.5\times 10^{-3},\;\text{initial}\;\text{condition}\;\text{at}\;t=0,\\ & \frac{\partial m(t)}{\partial x}=0,\;\text{boundary}\;\text{conditions}\;\text{for}\;x=0\;\text{and}\;x=1,\\ & \frac{\partial m(t)}{\partial y}=0,\;\text{boundary}\;\text{conditions}\;\text{for}\;y=\text{0}\;\text{and}\;y=1. \end{array} \end{array}$$ (37)

We set (10) in (34), (35) and (36) thus

$$\begin{array}{c} \begin{array}{cc} \underset{m,U}{\text{min}}J\left(m,U,t\right) & =\frac{1}{2}\int_{t_{0}}^{t_{f}}(m{\left(t\right)}^{T}m\left(t\right)+U{\left(t\right)}^{T}U\left(t\right))dt,\\ & s.t. \end{array} \end{array}$$ (38)

$$\begin{array}{c} \begin{array}{cc} \frac{\partial m(t)}{\partial t}-\frac{a}{\gamma }D_{m}\nabla^{2}m\left(t\right) & =(\lambda_{mfT}U\left(t\right)+\lambda_{mfG}\frac{G(t_{0})}{K_{G}+G\left(t_{0}\right)})f\left(t_{0}\right)-d_{m}m\left(t\right), \end{array} \end{array}$$ (39)

$$\begin{array}{c} \begin{array}{c} U\left(t_{0}\right)\leq U\left(t\right), \end{array} \end{array}$$ (40)

$$\begin{array}{c} \begin{array}{cc} & m\left(t_{0}\right)=8.5\times 10^{-3},\;\;\text{initial}\;\text{condition}\;\text{at}\;t_{0},\\ & \frac{\partial m(t)}{\partial x}=0,\;\text{boundary}\;\text{conditions}\;\text{for}\;x=0,1,\\ & \frac{\partial m(t)}{\partial y}=0,\;\text{boundary}\;\text{conditions}\;\text{for}\;y=0,1. \end{array} \end{array}$$ (41)

### 3.1 Pontryagin’s minimum principle

The minimization of the performance index *J* will be done using Pontryagin’s minimum principle [26, 30]. The extended Hamiltonian is

$$\begin{array}{c} \begin{array}{cc} \tilde{H}\left(m\left(t\right),U\left(t\right),\lambda \left(t\right),\mu ,t\right) & =m{\left(t\right)}^{T}m\left(t\right)+U{\left(t\right)}^{T}U\left(t\right)+\lambda^{T}\left(t\right)\left[Am\left(t\right)+BU\left(t\right)+b\right]\\ & +\mu (U\left(t_{0}\right)-U\left(t\right)), \end{array} \end{array}$$ (42)

where **λ**(*t*) is the vector of the Lagrange multipliers for (15) and ***μ*** is the Lagrange multiplier for (40) as follows

$$\begin{array}{c} \mu =\{\begin{array}{c} >0,\;\;U\left(t_{0}\right)=U\left(t\right),\\ =0,\;U\left(t_{0}\right)<U\left(t\right). \end{array} \end{array}$$ (43)

Define $\tilde{J}$ is as follows

$$\begin{array}{c} \begin{array}{cc} \tilde{J} & =\frac{1}{2}\int_{t_{0}}^{t_{f}}\left[\tilde{H}\left(m\left(t\right),U\left(t\right),\lambda \left(t\right)\mu ,t\right)-\lambda^{T}\left(t\right)\dot{m}\left(t\right)\right]dt. \end{array} \end{array}$$ (44)

A necessary condition for U* to minimize the performance index $\tilde{J}$ is

$$\begin{array}{c} \tilde{H}\left(m^{*},U^{*}\left(t\right),\lambda^{*}\left(t\right),\mu^{*}\left(t\right),t\right)\leq \tilde{H}\left(m^{*},U\left(t\right),\lambda^{*}\left(t\right),\mu^{*},t\right), \end{array}$$ (45)

for all admissible controls in *t* ∈ [*t*_0_, *t*_*f*_]. The vectors ***m*** and ***U*** must satisfy in the follows equations

$$\begin{array}{cc} & \frac{\partial \tilde{H}\left(m^{*}\left(t\right),U^{*}\left(t\right),\lambda^{*}\left(t\right),\mu^{*}\left(t\right),t\right)}{\partial m}=-{\dot{\lambda }}^{*}\left(t\right), \end{array}$$ (46)

$$\begin{array}{cc} & \frac{\partial \tilde{H}\left(m^{*}\left(t\right),U^{*}\left(t\right),\lambda^{*}\left(t\right),\mu^{*}\left(t\right),t\right)}{\partial \lambda }={\dot{m}}^{*}\left(t\right), \end{array}$$ (47)

$$\begin{array}{cc} & \tilde{H}\left(m^{*}\left(t\right),U^{*}\left(t\right),\lambda^{*}\left(t\right),\mu^{*}\left(t\right),t\right)\leq \tilde{H}\left(m^{*}\left(t\right),U\left(t\right),\lambda^{*}\left(t\right),\mu^{*}\left(t\right),t\right), \end{array}$$ (48)

$$\begin{array}{cc} & \mu^{*}(U\left(t_{0}\right)-U\left(t\right))=0. \end{array}$$ (49)

In this case, for $T_{GF}^{*}\left(t\right)$ to minimize the Hamiltonian equation. It is necessary that

$$\begin{array}{c} \frac{\partial \tilde{H}\left(m^{*}\left(t\right),U^{*}\left(t\right),\lambda^{*}\left(t\right),\mu^{*}\left(t\right),t\right)}{\partial U}=0. \end{array}$$ (50)

If Eq (50) is satisfied, and the matrix

$$\begin{array}{c} \frac{\partial^{2}\tilde{H}\left(m^{*}\left(t\right),U^{*}\left(t\right),\lambda^{*}\left(t\right),\mu^{*}\left(t\right),t\right)}{\partial U^{2}}, \end{array}$$ (51)

is positive definite, this is sufficient to guarantee that U*(*t*) causes $\tilde{H}$ to be a local minimum. Similar to Section 2.1.3, we are solved the optimal control problem (38)–(41). The following theorem holds for the third model problem (34)–(37).

*Theorm 3.1.* The optimal solution $T_{GF}^{*}\left(t\right)$ for the third model problem (34)–(37) exists. By solving the optimal control problem (38)–(41) and using Eq (10) the optimal transforming growth factor-*β* $\left(T_{GF}^{*}\left(t\right)\right)$ is in the following form:

$$\begin{array}{ccc} & \text{(i)}\;T_{GF}^{*}\left(t\right)=\frac{K_{T_{GF}}\left(K\left(t\right)m^{*}\left(t\right)+\rho \left(t\right)\right)}{1-K\left(t\right)m^{*}\left(t\right)-\rho \left(t\right)}\; & \text{for}\;\mu =0, \end{array}$$ (52)

$$\begin{array}{ccc} & \text{(ii)}\;T_{GF}^{*}\left(t\right)=T_{GF}\left(t_{0}\right)\;\; & \text{for}\;\mu >0. \end{array}$$ (53)

**Proof**: For existent of the optimal sultion see Theorem 2.1. To prove (i) and (ii) consider the following. From (49), one can show that in case (i) $\tilde{H}=H$ and $T_{GF}^{*}\left(t\right)$ is the same function as it is derived by (24) and (33). In the case (ii), from (49) and (10) it is obvious that in the all times between *t*_0_ = 0 up to *t*_*f*_ = 300 days $\frac{K_{T_{GF}}T_{GF}\left(t_{0}\right)}{1-T_{GF}\left(t_{0}\right)}=\frac{K_{T_{GF}}T_{GF}\left(t\right)}{1-T_{GF}\left(t\right)}$ thus $T_{GF}^{*}\left(t\right)=T_{GF}\left(t_{0}\right)$.

### 3.2 Numerical results

**Fig 6:**
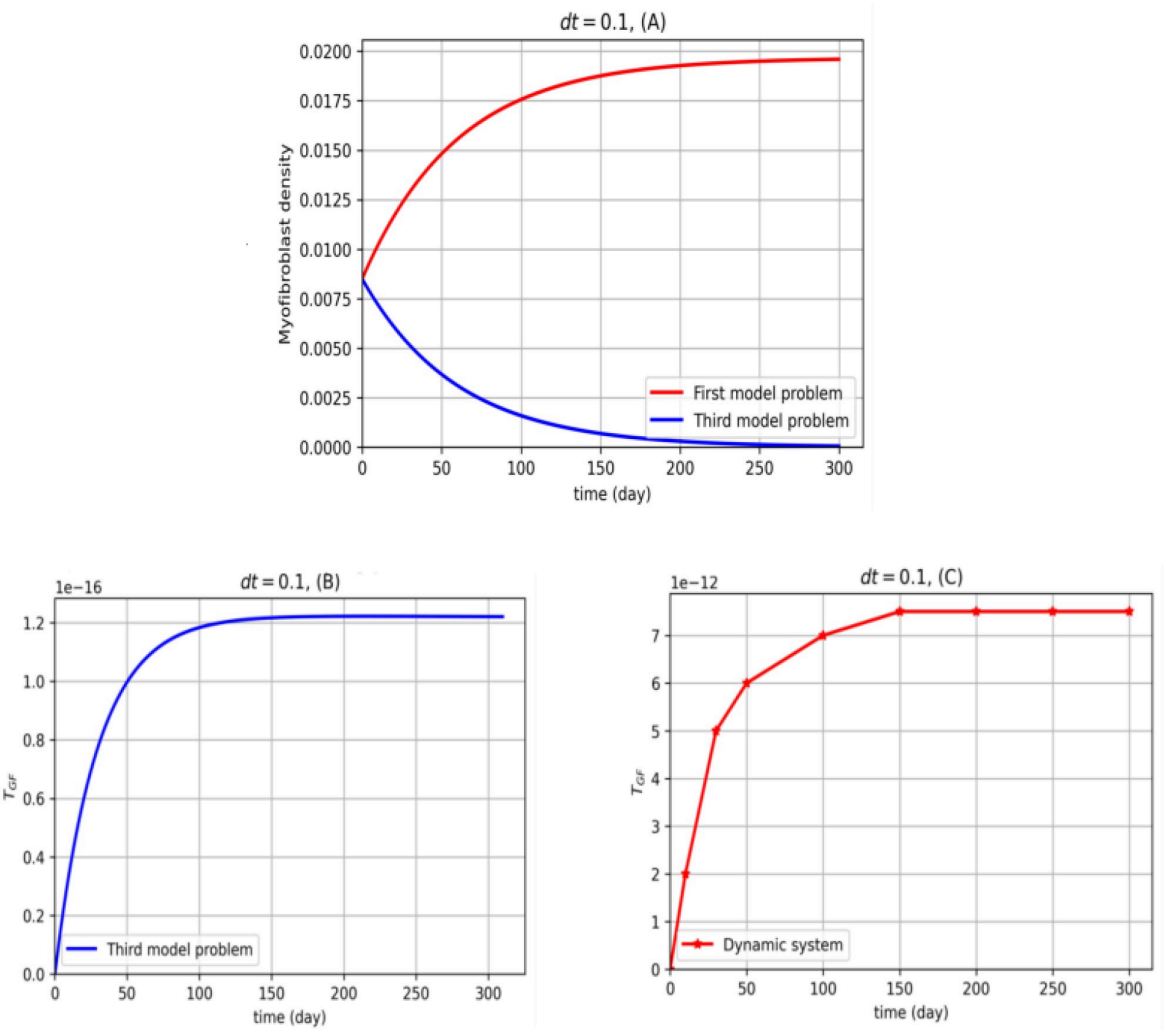
Improving second optimal control problem for myofibroblast diffusion. In (A), the third model problem (34)–(37) is solved by the technique proposed in this section (blue). The first model problem (dynamic system (8) and (9)) is solved by the central finite differences explicit method (red). In (B), an optimal control problem is depicted by solving the third model problem (blue). In (C), an optimal TGF-*β* function is calculated using the dynamical system in Ref. [20] (−⋆−). As it is shown in (B) the optimal TGF-*β* values are positive. Thus one finds out that numerical results are reasonable. In this regard, physicians in practice can prescribe anti-TGF-*β* drugs to restore TGF-*β* values to the optional values (an optimal strategy). This strategy can be applicable by prescribing anti-TGF-*β* drags. In solving the central finite differences explicit method all calculations are done for 36 nodes in the x-y plane.

In Fig 6, the numerical results for the third model problem are plotted. The Algorithm 2 is as follows:

- Step 1: We convert Eq (6) to Eq (15) using the discretization of second-order central finite differences.
- Step 2: We set the initial value in Table 1 for TGF-*β* control and myofibroblast state.
- Step 3: If ***μ*** = 0. We calculate TGF-*β* and myofibroblast state values in the same way as Algorithm 2.2.
- Step 4: If ***μ*** > 0. We set $T_{GF}^{*}\left(t\right)=T_{GF}\left(t_{0}\right)$.
- Step 5: We update the control and state in each iteration by using the values of the optimality system calculated in the previous iterations.
- Step 6: The procedure is continued iteratively till the convergence is achieved.

From Fig 6(B), by solving the third model problem during the time *t* = 0 to *t* = 300 days, it is derived that anti-TGF-*β* drugs must be used to restore TGF-*β* values to optimal values.

## 4 Novel model problem using myofibroblast and fibroblast

Here, we consider two dynamic equations in constraints including myofibroblast *m*(*t*) and fibroblast *f*(*t*).

### 4.1 Fourth model problem (fibroblast PDE)

We propose the fibroblast diffusion equation based on Fig 1 and Table 2 as follows

$$\begin{array}{c} \begin{array}{c} \frac{\partial f}{\partial t}-D_{f}\nabla^{2}f=\underset{source}{\underset{︸}{\lambda_{Ef}E_{0}}}+\underset{production}{\underset{︸}{\lambda_{fE}(\frac{T_{GFf}\left(t\right)}{K_{T_{GF}}+T_{GFf}\left(t\right)})\frac{E(t_{0})}{E\left(t_{0}\right)+K_{E}}}}-\underset{apoptosis}{\underset{︸}{d_{f}f\left(t\right)}}\\ -\underset{f\to m}{\underset{︸}{(\lambda_{mfG}\frac{G(t_{0})}{K_{G}+G\left(t_{0}\right)})f\left(t\right)}}-\underset{f\to T_{GF}}{\underset{︸}{\lambda_{T_{GF}f}\frac{E(t_{0})}{E\left(t_{0}\right)+K_{E}}f\left(t\right)}}-\underset{f\to \rho }{\underset{︸}{\lambda_{\rho f}f\left(t\right)}}. \end{array} \end{array}$$ (54)

**Table 2:** Parameters’ description.

Description		Value
*E*	density of activated AEC	*E*(*x*_0_, *y*_0_, *t*_0_) = 7.99 × 10^−1^ gcm^−3^ [19, 20]
*E*_0_	density of AEC	*E*_0_(*x*_0_, *y*_0_, *t*_0_) = 7.99 × 10^−1^ gcm^−3^ [19, 20]
*T*_*GFf*_	concentration of activated TGF-*β* in fibroblast	*T*_*GFf*_(*x*_0_, *y*_0_, *t*_0_) = 2.51 × 10^−12^gcm^−3^ [20]
*T*_*GFm*_	concentration of activated TGF-*β* in myofibroblast	*T*_*GFm*_(*x*_0_, *y*_0_, *t*_0_) = 2.51 × 10^−12^gcm^−3^ [20]
*ρ*	density of ECM	*ρ*(*x*_0_, *y*_0_, *t*_0_) = 3 × 10^−3^gcm^−3^ [19]
*d*_*f*_	death rate of fibroblasts	1.66 × 10^−2^*day*^−1^ [19, 20]
*D*_*f*_	the diffusion coefficient of fibroblasts	1.47 × 10^−6^*cm*^2^*day*^−1^ [19, 20]
λ_*fE*_	production rate of fibroblasts	5 × 10^−4^*day*^−1^ [19, 20]
λ_*ρf*_	activation rate of ECM due to fibroblasts	3 × 10^−3^*day*^−1^ [19, 20]
λ_*T*_*GF*_*f*_	production rate of TGF-*β* by fibroblast	7.5 × 10^−3^*day*^−1^ [19, 20]
λ_*Ef*_	activation rate of fibroblasts due to bFGF and TGF-*β*	2.5 × 10^−1^*gcm*^−3^ [19, 20]
*K*_*E*_	AEC saturation	10^−1^*gcm*^−3^ [19, 20]

The first term in Eq (54) is a source from E0 derived bFGF, which we take to be in the form λ_*Ef*_ *E*_0_. The growth factor bFGF is produced by AECs and activates fibroblasts [31]. Similar to [20] for simplicity, our model does not specifically include bFGF. Instead, represent it by *E*. The production of fibroblasts in healthy normal tissue depends on the density of AECs in homeostasis and is represented by the term λ_*Ef*_ *E*_0_. In IPF, there is additional production of fibroblasts by *E* is derived bFGF and *T*_*GF*_ [6, 19, 32] (the second term of the right-hand side of Eq (54)). Fibroblast transforms into myofibroblast by *T*_*GF*_ and concentration of PDGF [8–10, 33]. We write fibroblast transformation into myofibroblast by PDGF (the fourth term of the right-hand side of Eq (54)). *T*_*GF*_ is produced, and it is activated by fibroblasts and AECs [34, 35] (the fifth term of the right-hand side of Eq (54)). Fibroblast transformation into ECM [36] is represented by the last term on the right-hand side of Eq (54).

### 4.2 Fifth model problem

In the fifth model problem, we will use the fibroblast diffusion Eq (54). To write down the novel optimal control problem consisting of both fibroblast and myofibroblast diffusion equations as a dynamic system, one will write the state vector containing two variables, myofibroblast *m*(*t*) and fibroblast *f*(*t*).

$$\begin{array}{c} \begin{array}{c} \underset{m,f,T_{GFm},T_{GFf}}{\text{min}}\bar{J}\left(m\left(t\right),f\left(t\right),T_{GFm}\left(t\right),T_{GFf}\left(t\right),t\right)=\frac{1}{2}\int_{t_{0}}^{t_{f}}{\left(m\left(t\right)-f\left(t\right)\right)}^{T}\left(m\left(t\right)-f\left(t\right)\right)\\ +{\left(\frac{T_{GFm}\left(t\right)}{K_{T_{GF}}+T_{GFm}\left(t\right)}\right)}^{T}\frac{T_{GFm}\left(t\right)}{K_{T_{GF}}+T_{GFm}\left(t\right)}+{\left(\frac{T_{GFf}\left(t\right)}{K_{T_{GF}}+T_{GFf}\left(t\right)}\right)}^{T}\frac{T_{GFf}\left(t\right)}{K_{T_{GF}}+T_{GFf}\left(t\right)}dt, \end{array} \end{array}$$ (55)

$$\begin{array}{c} \begin{array}{cc} s.t.\\ \frac{\partial m(t)}{\partial t}-\frac{a}{\gamma }D_{m}\nabla^{2}m\left(t\right) & =(\lambda_{mfT}\frac{T_{GFm}\left(t\right)}{K_{T_{GF}}+T_{GFm}\left(t\right)}+\lambda_{mfG}\frac{G(t_{0})}{K_{G}+G\left(t_{0}\right)})f\left(t_{0}\right)\\ & -d_{m}m\left(t\right), \end{array} \end{array}$$ (56)

$$\begin{array}{c} \begin{array}{c} \frac{\partial f(t)}{\partial t}-\frac{a}{\gamma }D_{f}\nabla^{2}f\left(t\right)=\lambda_{Ef}E_{0}+\lambda_{fE}(\frac{T_{GFf}\left(t\right)}{K_{T_{GF}}+T_{GFf}\left(t\right)})\frac{E(t_{0})}{E\left(t_{0}\right)+K_{E}}-d_{f}f\left(t\right)\\ -(\lambda_{mfG}\frac{G(t_{0})}{K_{G}+G\left(t_{0}\right)})f\left(t\right)-\lambda_{T_{GF}f}\frac{E(t_{0})}{E\left(t_{0}\right)+K_{E}}f\left(t\right)-\lambda_{\rho f}f\left(t\right), \end{array} \end{array}$$ (57)

$$\begin{array}{cc} & \frac{K_{T_{GF}}T_{GFm}\left(t_{0}\right)}{1-T_{GFm}\left(t_{0}\right)}\leq \frac{K_{T_{GF}}T_{GFm}\left(t\right)}{1-T_{GFm}\left(t\right)}, \end{array}$$ (58)

$$\begin{array}{cc} & \frac{K_{T_{GF}}T_{GFf}\left(t_{0}\right)}{1-T_{GFf}\left(t_{0}\right)}\leq \frac{K_{T_{GF}}T_{GFf}\left(t\right)}{1-T_{GFf}\left(t\right)}, \end{array}$$ (59)

$$\begin{array}{c} \begin{array}{cc} & [\begin{array}{c} m(t_{0})\\ f(t_{0}) \end{array}]=[\begin{array}{c} 8.5\times 10^{-3}\\ 4.75\times 10^{-3} \end{array}],\;\text{initial}\;\text{condition}\;\text{at}\;t=0,\\ & [\begin{array}{c} \frac{\partial m}{\partial x}\\ \frac{\partial f}{\partial x} \end{array}]=[\begin{array}{c} 0\\ 0 \end{array}],\;\text{boundary}\;\text{conditions}\;\text{for}\;x=0,1,\\ & [\begin{array}{c} \frac{\partial m}{\partial y}\\ \frac{\partial f}{\partial y} \end{array}]=[\begin{array}{c} 0\\ 0 \end{array}],\;\text{boundary}\;\text{conditions}\;\text{for}\;y=0,1. \end{array} \end{array}$$ (60)

Similar to Section 2.1.2 and Section 3, we set

$$\begin{array}{c} U_{m}\left(t\right)=\frac{T_{GFm}\left(t\right)}{K_{T_{GF}}+T_{GFm}\left(t\right)}, \end{array}$$ (61)

and

$$\begin{array}{c} U_{f}\left(t\right)=\frac{T_{GFf}\left(t\right)}{K_{T_{GF}}+T_{GFf}\left(t\right)}, \end{array}$$ (62)

thus

$$\begin{array}{c} T_{GFm}\left(t\right)=\frac{K_{T_{GF}}U_{m}\left(t\right)}{1-U_{m}\left(t\right)}, \end{array}$$ (63)

$$\begin{array}{c} T_{GFf}\left(t\right)=\frac{K_{T_{GF}}U_{f}\left(t\right)}{1-U_{f}\left(t\right)}. \end{array}$$ (64)

We use (61) and (62) in (55)–(59). Moreover, we have

$$\begin{array}{c} \begin{array}{cc} \underset{m,f,U}{\text{min}}\bar{J}\left(m\left(t\right),f\left(t\right),U_{m}\left(t\right),U_{f}\left(t\right),t\right) & =\frac{1}{2}\int_{t_{0}}^{t_{f}}{\left(m\left(t\right)-f\left(t\right)\right)}^{T}\left(m\left(t\right)-f\left(t\right)\right)\\ & +U_{m}{\left(t\right)}^{T}U_{m}\left(t\right)+U_{f}{\left(t\right)}^{T}U_{f}\left(t\right)dt,\\ & s.t. \end{array} \end{array}$$ (65)

$$\begin{array}{c} \begin{array}{c} \frac{\partial m(t)}{\partial t}-\frac{a}{\gamma }D_{m}\nabla^{2}m\left(t\right)=(\lambda_{mfT}U_{m}\left(t\right)+\lambda_{mfG}\frac{G(t_{0})}{K_{G}+G\left(t_{0}\right)})f\left(t_{0}\right)-d_{m}m\left(t\right), \end{array} \end{array}$$ (66)

$$\begin{array}{c} \begin{array}{cc} & \frac{\partial f(t)}{\partial t}-\frac{a}{\gamma }D_{f}\nabla^{2}f\left(t\right)=\lambda_{Ef}E_{0}+(\lambda_{fE}\frac{E(t_{0})}{E\left(t_{0}\right)+K_{E}})U_{f}\left(t\right)-d_{f}f\left(t\right)\\ & -(\lambda_{mfG}\frac{G(t_{0})}{K_{G}+G\left(t_{0}\right)})f\left(t\right)-(\lambda_{T_{GF}f}\frac{E(t_{0})}{E\left(t_{0}\right)+K_{E}})f\left(t\right)-\lambda_{\rho f}f\left(t\right), \end{array} \end{array}$$ (67)

$$\begin{array}{c} \begin{array}{cc} & U_{m}\left(t_{0}\right)\leq U_{m}\left(t\right),\\ & U_{f}\left(t_{0}\right)\leq U_{f}\left(t\right), \end{array} \end{array}$$ (68)

$$\begin{array}{c} \begin{array}{cc} & [\begin{array}{c} m(t_{0})\\ f(t_{0}) \end{array}]=[\begin{array}{c} 8.5\times 10^{-3}\\ 4.75\times 10^{-3} \end{array}],\;\text{initial}\;\text{condition}\;\text{at}\;t=0,\\ & [\begin{array}{c} \frac{\partial m}{\partial x}\\ \frac{\partial f}{\partial x} \end{array}]=[\begin{array}{c} 0\\ 0 \end{array}],\;\text{boundary}\;\text{conditions}\;\text{for}\;x=0,1,\\ & [\begin{array}{c} \frac{\partial m}{\partial y}\\ \frac{\partial f}{\partial y} \end{array}]=[\begin{array}{c} 0\\ 0 \end{array}],\;\text{boundary}\;\text{conditions}\;\text{for}\;y=0,1. \end{array} \end{array}$$ (69)

Eqs (66) and (67) are in a matrix form as follows

$$\begin{array}{c} \begin{array}{cc} & \frac{dm}{dt}=A_{m}m\left(t\right)+B_{m}U_{m}\left(t\right)+b_{m},\\ & \frac{df}{dt}=A_{f}f\left(t\right)+B_{f}U_{f}\left(t\right)+b_{f}, \end{array} \end{array}$$ (70)

where block matrices have the following forms

*A*_*m*_ = *A*, *B*_*m*_ = *B* and ***b***_***m***_ = ***b*** (see Section 2.1.2). Moreover, we propose

$$\begin{array}{cc} A_{f} & ={\left[\begin{array}{cccccc} G_{f} & L_{f}^{T} & O & O & \cdots & O\\ L_{f} & G_{f} & L_{f}^{T} & O & \cdots & O\\ O & L_{f} & G_{f} & L_{f}^{T} & \cdots & O\\ \vdots & \; & \ddots & \ddots & \ddots & \vdots \\ O & O & \cdots & O & L_{f} & G_{f} \end{array}\right]}_{{\left(n-1\right)}^{2}\times {\left(n-1\right)}^{2}}, \end{array}$$

$$\begin{array}{cc} G_{f} & ={\left[\begin{array}{ccccc} -2r_{f}-\alpha & r_{f} & 0 & \cdots & 0\\ r_{f} & -2r_{f}-\alpha & r_{f} & \cdots & 0\\ 0 & r_{f} & -2r_{f}-\alpha & \cdots & 0\\ \vdots & \ddots & \ddots & \ddots & r_{f}\\ 0 & \cdots & 0 & r_{f} & -2r_{f}-\alpha \end{array}\right]}_{\left(n-1)\times (n-1\right)}, \end{array}$$

$$\begin{array}{cc} L_{f} & ={\left[\begin{array}{ccccc} 0 & 0 & 0 & \cdots & 0\\ r_{f} & 0 & 0 & \cdots & 0\\ 0 & r_{f} & 0 & \cdots & 0\\ \vdots & \ddots & \ddots & \ddots & \vdots \\ 0 & \cdots & 0 & r_{f} & 0 \end{array}\right]}_{\left(n-1)\times (n-1\right)}, \end{array}$$

in which, $r_{f}=\frac{a(D_{f}\times dx^{2})}{\gamma }$, $\alpha =\lambda_{mfG}\frac{G(t_{0})}{K_{G}+G\left(t_{0}\right)}+\lambda_{T_{GF}f}\frac{E(t_{0})}{E\left(t_{0}\right)+K_{E}}+\lambda_{\rho f}+d_{f}$ and

$$\begin{array}{c} B_{f}={\left[\begin{array}{ccccc} \theta_{f}I & I & 0 & \cdots & 0\\ I & \theta_{f}I & I & \cdots & 0\\ 0 & I & \theta_{f}I & \cdots & 0\\ \vdots & \ddots & \ddots & \ddots & I\\ 0 & \cdots & 0 & I & \theta_{f}I \end{array}\right]}_{{\left(n-1\right)}^{2}\times {\left(n-1\right)}^{2}}, \end{array}$$

in which, $\theta_{f}=\lambda_{fE}\frac{E(t_{0})}{E\left(t_{0}\right)+K_{E}}$.

$$\begin{array}{c} \begin{array}{c} b_{f}={\left[\begin{array}{c} b_{f1}\\ b_{f2}\\ \vdots \\ b_{fn} \end{array}\right]}_{{\left(n-1\right)}^{2}\times 1}b_{fj}=r{\left[\begin{array}{c} f_{1,j}\left(t\right)\\ 0\\ \vdots \\ 0\\ f_{n,j}\left(t\right) \end{array}\right]}_{(n-1)\times 1}+c_{f}{\left[\begin{array}{c} 1\\ 1\\ \vdots \\ 1\\ 1 \end{array}\right]}_{(n-1)\times 1}, \end{array} \end{array}$$ (71)

in which, *c*_*f*_ = λ_*Ef*_ *E*_0_ and *f*_*i*,*j*_(*t*_0_) = 4.75 × 10^−3^. Moreover,

$$\begin{array}{c} \begin{array}{c} f\left(t\right)={\left[\begin{array}{c} f_{1}\left(t\right)\\ f_{2}\left(t\right)\\ \vdots \\ f_{n}\left(t\right) \end{array}\right]}_{{\left(n-1\right)}^{2}\times 1}f_{j}\left(t\right)={\left[\begin{array}{c} f_{1,j}\left(t\right)\\ f_{2,j}\left(t\right)\\ \vdots \\ f_{n,j}\left(t\right) \end{array}\right]}_{(n-1)\times 1}. \end{array} \end{array}$$ (72)

***b***_***f*** ***j***_ is a column vector of zeros and known boundary values which are added with a fixed value of *c*_*f*_.

### 4.3 Solution for the fifth model problem

The novel Hamiltonian function is

$$\begin{array}{c} \begin{array}{cc} & \bar{H}\left(m\left(t\right),f\left(t\right),U_{m}\left(t\right),U_{f}\left(t\right),\lambda_{1}\left(t\right),\lambda_{2}\left(t\right),\mu_{m},\mu_{f},t\right)={\left(m\left(t\right)-f\left(t\right)\right)}^{T}\left(m\left(t\right)-f\left(t\right)\right)\\ & +U_{m}{\left(t\right)}^{T}U_{m}\left(t\right)+U_{f}{\left(t\right)}^{T}U_{f}\left(t\right)+\lambda_{1}^{T}\left(t\right)\left[A_{m}m\left(t\right)+B_{m}U_{m}\left(t\right)+b_{m}\right]\\ & +\lambda_{2}^{T}\left(t\right)\left[A_{f}f\left(t\right)+B_{f}U_{f}\left(t\right)+b_{f}\right]+\mu_{m}(U_{m}\left(t_{0}\right)-U_{m}\left(t\right))+\mu_{f}(U_{f}\left(t_{0}\right)-U_{f}\left(t\right)), \end{array} \end{array}$$ (73)

where **λ**_**1**_ and **λ**_**2**_ are vectors of the Lagrange multipliers for (70), ***μ***_***m***_ and ***μ***_***f***_ are Lagrange multipliers for (68) as follows

$$\begin{array}{c} \mu_{m}=\{\begin{array}{c} >0,\;\;U_{m}\left(t_{0}\right)=U_{m}\left(t\right),\\ =0,\;U_{m}\left(t_{0}\right)<U_{m}\left(t\right), \end{array} \end{array}$$ (74)

$$\begin{array}{c} \mu_{f}=\{\begin{array}{c} >0,\;\;U_{f}\left(t_{0}\right)=U_{f}\left(t\right),\\ =0,\;U_{f}\left(t_{0}\right)<U_{f}\left(t\right). \end{array} \end{array}$$ (75)

The performance index $\bar{J}$ is

$$\begin{array}{c} \begin{array}{cc} & \bar{J}=\frac{1}{2}\int_{t_{0}}^{t_{f}}[\bar{H}\left(m\left(t\right),f\left(t\right),U_{m}\left(t\right),U_{f}\left(t\right),\lambda_{1}\left(t\right),\lambda_{2}\left(t\right),\mu_{m}\left(t\right),\mu_{f}\left(t\right),t\right)-\lambda_{1}^{T}\left(t\right)\dot{m}\left(t\right)\\ & -\lambda_{2}^{T}\left(t\right)\dot{f}\left(t\right)]dt. \end{array} \end{array}$$ (76)

Necessary conditions for $U_{m}^{*}$ and $U_{f}^{*}$ to minimize the performance index $\bar{J}$ is

$$\begin{array}{c} \bar{H}\left(m^{*},f^{*},U_{m}^{*},U_{f}^{*},\lambda_{1}^{*},\lambda_{2}^{*},\mu_{m}^{*},\mu_{f}^{*},t\right)\leq \bar{H}\left(m^{*},f^{*},U_{m},U_{f}^{*},\lambda_{1}^{*},\lambda_{2}^{*},\mu_{m}^{*},\mu_{f}^{*},t\right), \end{array}$$

and

$$\begin{array}{c} \bar{H}\left(m^{*},f^{*},U_{m}^{*},U_{f}^{*},\lambda_{1}^{*},\lambda_{2}^{*},\mu_{m}^{*},\mu_{f}^{*},t\right)\leq \bar{H}\left(m^{*},f^{*},U_{m}^{*},U_{f},\lambda_{1}^{*},\lambda_{2}^{*},\mu_{m}^{*},\mu_{f}^{*},t\right), \end{array}$$

for all admissible controls in *t* ∈ [*t*_0_, *t*_*f*_]. The vectors ***m***, ***f***, ***U***_***m***_ and ***U***_***f***_ must satisfy the equations

$$\begin{array}{cc} & \frac{\partial \bar{H}\left(m^{*},f^{*},U_{m}^{*},U_{f}^{*},\lambda_{1}^{*},\lambda_{2}^{*},\mu_{m}^{*},\mu_{f}^{*},t\right)}{\partial m}=-{\dot{\lambda }}_{1}^{*}, \end{array}$$ (77)

$$\begin{array}{cc} & \frac{\partial \bar{H}\left(m^{*},f^{*},U_{m}^{*},U_{f}^{*},\lambda_{1}^{*},\lambda_{2}^{*},\mu_{m}^{*},\mu_{f}^{*},t\right)}{\partial f}=-{\dot{\lambda }}_{2}^{*}, \end{array}$$ (78)

$$\begin{array}{cc} & \frac{\partial \bar{H}\left(m^{*},f^{*},U_{m}^{*},U_{f}^{*},\lambda_{1}^{*},\lambda_{2}^{*},\mu_{m}^{*},\mu_{f}^{*},t\right)}{\partial \lambda_{1}}={\dot{m}}^{*}, \end{array}$$ (79)

$$\begin{array}{cc} & \frac{\partial \bar{H}\left(m^{*},f^{*},U_{m}^{*},U_{f}^{*},\lambda_{1}^{*},\lambda_{2}^{*},\mu_{m}^{*},\mu_{f}^{*},t\right)}{\partial \lambda_{2}}={\dot{f}}^{*}, \end{array}$$ (80)

$$\begin{array}{cc} & \bar{H}\left(m^{*},f^{*},U_{m}^{*},U_{f}^{*},\lambda_{1}^{*},\lambda_{2}^{*},\mu_{m}^{*},\mu_{f}^{*},t\right)\leq \bar{H}\left(m^{*},f^{*},U_{m},U_{f}^{*},\lambda_{1}^{*},\lambda_{2}^{*},\mu_{m}^{*},\mu_{f}^{*},t\right), \end{array}$$ (81)

$$\begin{array}{cc} & \bar{H}\left(m^{*},f^{*},U_{m}^{*},U_{f}^{*},\lambda_{1}^{*},\lambda_{2}^{*},\mu_{m}^{*},\mu_{f}^{*},t\right)\leq \bar{H}\left(m^{*},f^{*},U_{m}^{*},U_{f},\lambda_{1}^{*},\lambda_{2}^{*},\mu_{m}^{*},\mu_{f}^{*},t\right), \end{array}$$ (82)

$$\begin{array}{cc} & \mu_{m}^{*}(U_{m}\left(t_{0}\right)-U_{m}\left(t\right))=0, \end{array}$$ (83)

$$\begin{array}{cc} & \mu_{f}^{*}(U_{f}\left(t_{0}\right)-U_{f}\left(t\right))=0. \end{array}$$ (84)

In this case, for $T_{GFm}^{*}\left(t\right)$ and $T_{GFf}^{*}\left(t\right)$ to minimize the Hamiltonian equation it is necessary that

$$\begin{array}{c} \begin{array}{c} \frac{\partial \bar{H}\left(m^{*},f^{*},U_{m}^{*},U_{f}^{*},\lambda_{1}^{*},\lambda_{2}^{*},\mu_{m}^{*},\mu_{f}^{*},t\right)}{\partial U_{m}}=0,\\ \frac{\partial \bar{H}\left(m^{*},f^{*},U_{m}^{*},U_{f}^{*},\lambda_{1}^{*},\lambda_{2}^{*},\mu_{m}^{*},\mu_{f}^{*},t\right)}{\partial U_{f}}=0. \end{array} \end{array}$$ (85)

From (85), we have that

$$\begin{array}{c} \begin{array}{cc} & U_{m}^{*}\left(t\right)=-B_{m}^{T}\lambda_{1}^{*}\left(t\right),\\ & U_{f}^{*}\left(t\right)=-B_{f}^{T}\lambda_{2}^{*}\left(t\right). \end{array} \end{array}$$ (86)

A way to find the optimal control is linear feedback form; that is, to look for function *K*_*m*_(*t*), *K*_*f*_(*t*), ***ρ***_***m***_(*t*) and ***ρ***_***f***_(*t*) for which

$$\begin{array}{c} \begin{array}{cc} & U_{m}^{*}\left(t\right)=K_{m}\left(t\right)m^{*}\left(t\right)+\rho_{m}\left(t\right),\\ & U_{f}^{*}\left(t\right)=K_{f}\left(t\right)f^{*}\left(t\right)+\rho_{f}\left(t\right). \end{array} \end{array}$$ (87)

For the unknowns ***ρ***_***m***_(*t*), ***ρ***_***f***_(*t*), *K*_*m*_(*t*) and *K*_*f*_(*t*) as the feedback matrices. We assume that the vector of lagrange multiplier $\lambda_{1}^{*}\left(t\right)$ is linear in m*(*t*) and the vector of lagrange multiplier $\lambda_{2}^{*}\left(t\right)$ is linear in f*(*t*), i.e.

$$\begin{array}{c} \lambda_{1}^{*}\left(t\right)=p_{m}\left(t\right)m^{*}\left(t\right)-b_{m}\eta_{m}\left(t\right), \end{array}$$ (88)

$$\begin{array}{c} \lambda_{2}^{*}\left(t\right)=p_{f}\left(t\right)f^{*}\left(t\right)-b_{f}\eta_{f}\left(t\right), \end{array}$$ (89)

for the unknowns ***p***_***m***_(*t*), ***p***_***f***_(*t*), ***η***_***m***_(*t*) and ***η***_***f***_(*t*), if we substitute Eqs (88) and (89) in Eq (86), we have

$$\begin{array}{c} \begin{array}{cc} & U_{m}^{*}\left(t\right)=-B_{m}^{T}p_{m}\left(t\right)m^{*}\left(t\right)+B_{m}^{T}b_{m}\eta_{m}\left(t\right),\\ & U_{f}^{*}\left(t\right)=-B_{f}^{T}p_{f}\left(t\right)f^{*}\left(t\right)+B_{f}^{T}b_{f}\eta_{f}\left(t\right). \end{array} \end{array}$$ (90)

By comparing (90) and (87), we have

$$\begin{array}{cc} & K_{m}\left(t\right)=-B_{m}^{T}p_{m}\left(t\right),\;K_{f}\left(t\right)=-B_{f}^{T}p_{f}\left(t\right),\;\rho_{m}\left(t\right)=B_{m}^{T}b_{m}\eta_{m}\left(t\right)\;and \end{array}$$ (91)

$$\begin{array}{cc} & \rho_{f}\left(t\right)=B_{f}^{T}b_{f}\eta_{f}\left(t\right). \end{array}$$ (92)

By substitute Eq (90) in Eq (70), we have

$$\begin{array}{c} \begin{array}{c} {\dot{m}}^{*}\left(t\right)=A_{m}m^{*}\left(t\right)+B_{m}\left(-B_{m}^{T}p_{m}\left(t\right)m^{*}\left(t\right)+B_{m}^{T}b_{m}\eta_{m}\left(t\right)\right)+b_{m}, \end{array} \end{array}$$ (93)

and

$$\begin{array}{c} \begin{array}{c} {\dot{f}}^{*}\left(t\right)=A_{f}f^{*}\left(t\right)+B_{f}\left(-B_{f}^{T}p_{f}\left(t\right)f^{*}\left(t\right)+B_{f}^{T}b_{f}\eta_{f}\left(t\right)\right)+b_{f}. \end{array} \end{array}$$ (94)

Differentiate Eq (88) and use (77), and differentiate Eq (89) and use (78), we have

$$\begin{array}{c} \begin{array}{c} {\dot{\lambda }}_{1}^{*}\left(t\right)=p_{m}\left(t\right)m^{*}\left(t\right)+p_{m}\left(t\right){\dot{m}}^{*}\left(t\right)-b_{m}{\dot{\eta }}_{m}\left(t\right)=-m^{*}\left(t\right)-A_{m}^{T}\lambda_{1}^{*}\left(t\right), \end{array} \end{array}$$ (95)

and

$$\begin{array}{c} \begin{array}{c} {\dot{\lambda }}_{2}^{*}\left(t\right)=p_{f}\left(t\right)m^{*}\left(t\right)+p_{f}\left(t\right){\dot{f}}^{*}\left(t\right)-b_{f}{\dot{\eta }}_{f}\left(t\right)=-f^{*}\left(t\right)-A_{f}^{T}\lambda_{2}^{*}\left(t\right). \end{array} \end{array}$$ (96)

By substituting (93) in Eq (95) and using Eq (88), and Eq (94) in Eq (96) and using (89), we arrive at the following two relations

$$\begin{array}{c} \begin{array}{cc} m^{*}\left(t\right)({\dot{p}}_{m}\left(t\right)+p_{m}\left(t\right)A_{m}+A_{m}^{T}p_{m}\left(t\right)-p_{m}\left(t\right)B_{m}B_{m}^{T}p_{m}\left(t\right)+I) & +\\ b_{m}(-{\dot{\eta }}_{m}\left(t\right)-\eta_{m}\left(t\right)\left(A_{m}-B_{m}^{T}p_{m}\left(t\right)B_{m}\right)+p_{m}\left(t\right)) & =0, \end{array} \end{array}$$ (97)

and

$$\begin{array}{c} \begin{array}{cc} f^{*}\left(t\right)({\dot{p}}_{f}\left(t\right)+p_{f}\left(t\right)A_{f}+A_{f}^{T}p_{f}\left(t\right)-p_{f}\left(t\right)B_{f}B_{f}^{T}p_{f}\left(t\right)+I) & +\\ b_{f}(-{\dot{\eta }}_{f}\left(t\right)-\eta_{f}\left(t\right)\left(A_{f}-B_{f}^{T}p_{f}\left(t\right)B_{f}\right)+p_{f}\left(t\right)) & =0. \end{array} \end{array}$$ (98)

For Eq (97), m*(*t*) and ***b***_***m***_ are not zero thus the coefficient of m*(*t*) and the second term in Eq (97) must simultaneously be equal to zero. This reduces Eq (97) to the following two differential equations:

$$\begin{array}{c} {\dot{p}}_{m}\left(t\right)+p_{m}\left(t\right)A_{m}+A_{m}^{T}p_{m}\left(t\right)-p_{m}\left(t\right)B_{m}B_{m}^{T}p_{m}\left(t\right)=-I, \end{array}$$ (99)

by calculating ***p***_***m***_(*t*) from (99), one calculat ***η***_***m***_(*t*) by (100)

$$\begin{array}{c} {\dot{\eta }}_{m}\left(t\right)+\eta_{m}\left(t\right)\left(A_{m}-B_{m}^{T}p_{m}\left(t\right)B_{m}\right)=p_{m}\left(t\right). \end{array}$$ (100)

Similarly, For Eq (98), f*(*t*) and ***b***_***f***_ are not zero. The coefficient of f*(*t*) and the second term in Eq (98) must simultaneously be equal to zero. This reduces Eq (98) to the following two differential equations:

$$\begin{array}{c} \dot{p_{f}}\left(t\right)+p_{f}\left(t\right)A_{f}+A_{f}^{T}p_{f}\left(t\right)-p_{f}\left(t\right)B_{f}B_{f}^{T}p_{f}\left(t\right)=-I, \end{array}$$ (101)

by calculating ***p***_***f***_(*t*) from (101), one calculat ***η***_***f***_(*t*) by (102)

$$\begin{array}{c} \dot{\eta_{f}}\left(t\right)+\eta_{f}\left(t\right)\left(A_{f}-B_{f}^{T}p_{f}\left(t\right)B_{f}\right)=p_{f}\left(t\right). \end{array}$$ (102)

*Theorem 4.1.* A minimum ${\bar{J}}^{*}$ exists if and only if solutions ***p***_***m***_(*t*) and ***p***_***f***_(*t*) of the Riccati Eqs (99) and (101) exist, are bounded, and are positive definite for all *t* < *t*_*f*_. In this case, the minimum performance index ${\bar{J}}^{*}$ becomes

$$\begin{array}{c} {\bar{J}}^{*}=\frac{1}{2}(m{*}^{T}\left(t_{0}\right)p_{m}\left(t_{0}\right)m^{*}\left(t_{0}\right)-f{*}^{T}\left(t_{0}\right)p_{f}\left(t_{0}\right)f^{*}\left(t_{0}\right)). \end{array}$$ (103)

**Proof**: See Section 4.3 and Theorem 2.1.3.

*Proposition 4.1.* In practice, the optimal control problem (65)–(69) is solved and the optimal value is calculated by Eq (87). Using Eqs (61), (62) and (87), the optimal control problem (55)–(60) can be solved and its optimal value is as follows

$$\begin{array}{ccc} & \text{(I)}\;T_{GFm}^{*}\left(t\right)=\frac{K_{T_{GF}}\left(K\left(t\right)m^{*}\left(t\right)+\rho_{m}\left(t\right)\right)}{1-K\left(t\right)m^{*}\left(t\right)-\rho_{m}\left(t\right)}\; & \text{for}\;\mu_{m}=0, \end{array}$$ (104)

$$\begin{array}{ccc} & \text{(II)}\;T_{GFm}^{*}\left(t\right)=T_{GFm}\left(t_{0}\right)\;\; & \text{for}\;\mu_{m}>0, \end{array}$$ (105)

$$\begin{array}{ccc} & \text{(III)}\;T_{GFf}^{*}\left(t\right)=\frac{K_{T_{GF}}\left(K\left(t\right)f^{*}\left(t\right)+\rho_{f}\left(t\right)\right)}{1-K\left(t\right)f^{*}\left(t\right)-\rho_{f}\left(t\right)}\; & \text{for}\;\mu_{f}=0, \end{array}$$ (106)

$$\begin{array}{ccc} & \text{(IV)}\;T_{GFf}^{*}\left(t\right)=T_{GFf}\left(t_{0}\right)\;\; & \text{for}\;\mu_{f}>0. \end{array}$$ (107)

**Fig 7:**
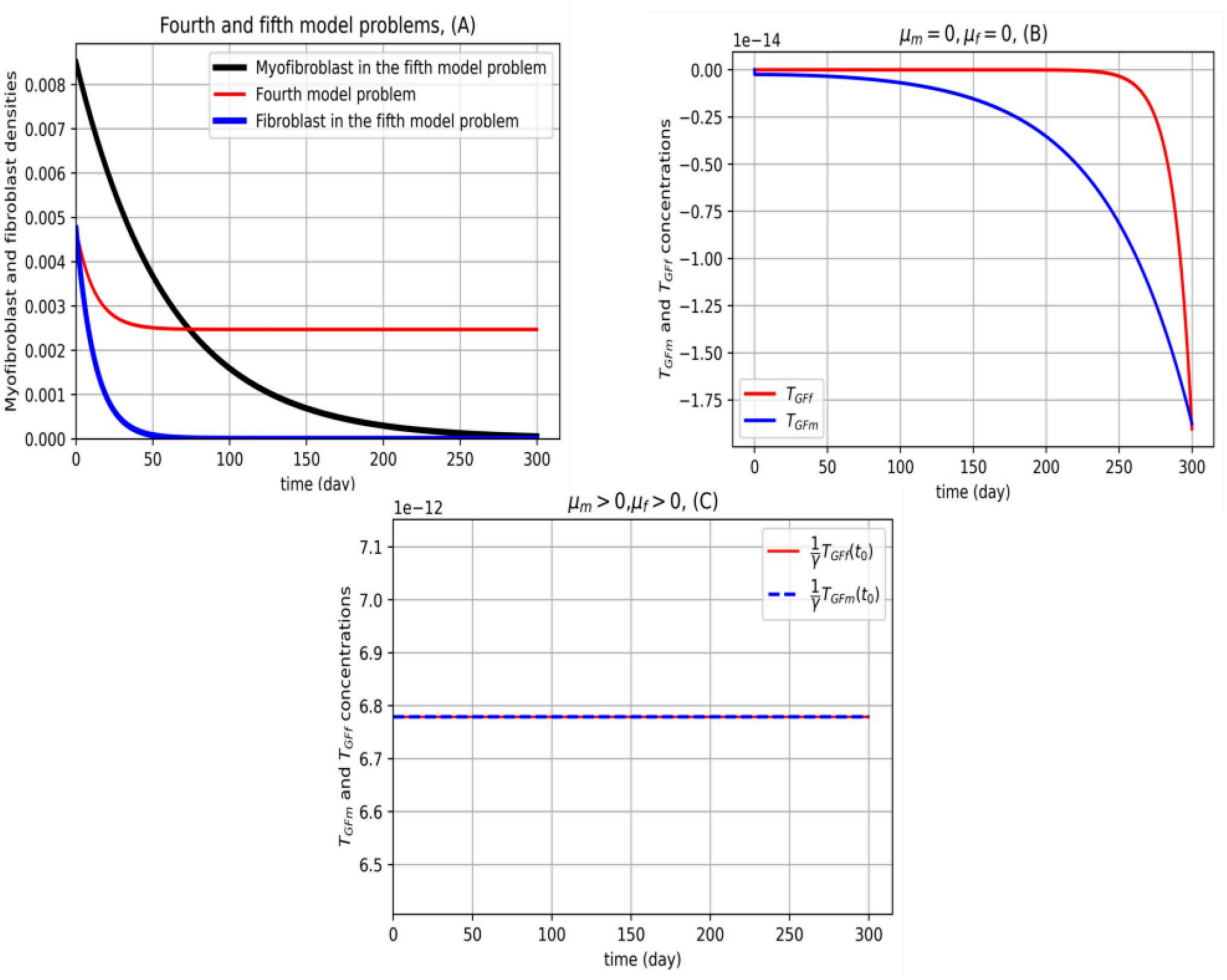
Novel model problem using myofibroblast and fibroblast for 36 nodes. In (A), the fifth model problem (55)–(60) is solved by the technique proposed in this section (black color for myofibroblast and blue color for fibroblast). The fourth model problem for the fibroblast dynamical system (fibroblast PDE (57)) is solved (red). (B) show the optimal constraint values of *T*_*GFm*_ and *T*_*GFf*_ concentrations. Since these two functions are not passive, one understands that in the real word problem, constraints (58) and (59) should be considered inactive. Thus by considering the fifth model problem, we are allowed just consider the active constraints (58) and (59). Therefore solution for TGF-*β* for myofibroblast and fibroblast in (B) is not acceptable. Note that *μ*_*m*_ and *μ*_*f*_ are the Lagrange coefficient of constraints (58) and (59) in the extended Hamiltonian equation. In (C), we assume that constraints (58) and (59) are active thus *T*_*GFm*_ and *T*_*GFf*_ remain constant over time. Overall from (A), it is observed that in 240 days myofibroblasts, and in 50 days, fibroblasts vanish when TGF-*β* concentration for both fibroblast and myofibrils should be assigned constant and taking into account that only *γ*-fraction of the space is occupied by tissue, the value $\frac{1}{\gamma }T_{GF}$ coincides with value of *T*_*GF*_ as computed in [18] equal to 6.77 × 10^−12^. This strategy can be applicable by prescribing anti-TGF-*β* drags. All calculations are done for 36 nodes in the x-y plane.

From Eqs (58) and (59), we have four cases. Case 1 is included Eqs (104) and (106), Case 2 includes Eqs (104) and (105), Case 3 includes Eqs (105) and (106) and Case 4 includes Eqs (105) and (107). For Cases 1 and 2, and 3, the optimal TGF-*β* is negative. Cases 1 and 4 are plotted in Fig 7(B) and 7(C) respectively. As is shown in Fig 7(C), the only accepting case is Case 4.

### 4.4 Numerical results

The Algorithm 3 is as follows:

- Step 1: We convert PDEs (56) and (57) into the system of ordinary Eq (70).
- Step 2: We set the initial values in Table 2 for control and myofibroblast and fibroblast states.
- Step 3: If ***μ***_***m***_ = 0 and ***μ***_***f***_ = 0. We solve Eqs (99)–(102).
- Step 4: Find ***T***_***G*** ***F*** ***m***_ and ***T***_***G*** ***F*** ***f***_ using the previous step and Eqs (63) and (64).
- Step 4: If ***μ***_***m***_ > 0, ***μ***_***f***_ > 0 or ***μ***_***m***_ = 0, ***μ***_***f***_ > 0 or ***μ***_***m***_ > 0, ***μ***_***f***_ = 0. We set $T_{GFm}^{*}\left(t\right)=T_{GFm}\left(t_{0}\right)$ and $T_{GFf}^{*}\left(t\right)=T_{GFf}\left(t_{0}\right)$.
- Step 5: We update the control and states in each iteration by using the values of the optimality system obtained in the previous iterations.
- Step 6: The procedure is continued iteratively till the convergence is achieved.

**Fig 8:**
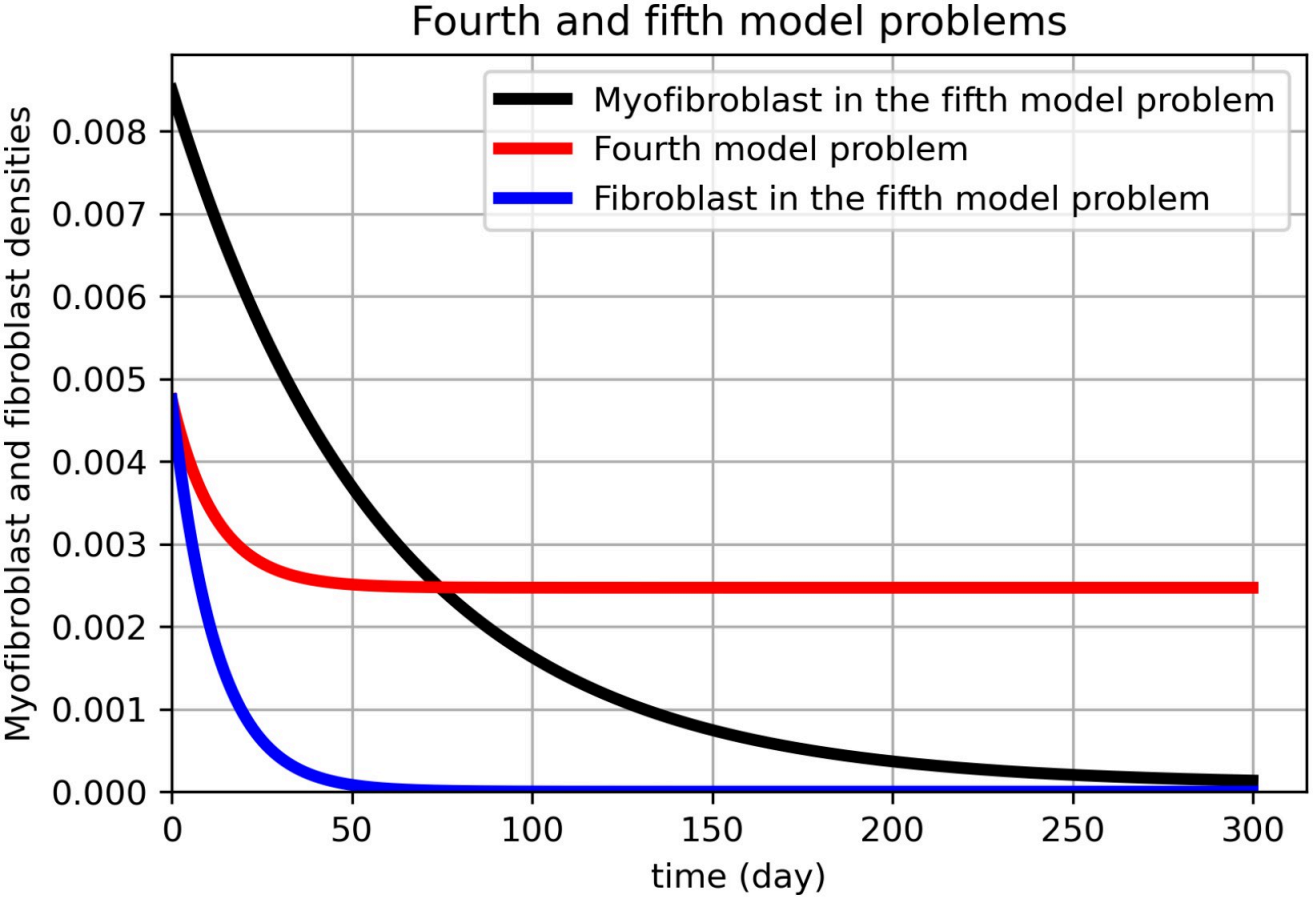
Novel model problem using myofibroblast and fibroblast for 64 nodes. In Fig 8, the fifth model problem (55)–(60) is solved by the technique proposed in this section with 64 nodes for the finite difference method (black color for myofibroblast and blue color for fibroblast). The fourth model problem for the fibroblast dynamical system (fibroblast PDE (57)) is solved (red). We compare Fig 7. (A) for 36 nodes with Fig 8 that is plotted for 64 nodes in the x-y plane. The graphs are almost the same. However, as it is shown in Fig 8, myofibroblast densities vanish in 290 days, while fibroblast density vanishes in 50 days. Since more discretization nodes give more accurate numerical solutions, we believe this solution is more reasonable. Thus the optimal strategy can get from calculations done by 64 nodes.

Fig 7 shows the results of solving the novel optimal control problem with 36 nodes for the finite difference discretization method. As shown in Fig 8, the number of nodes has been increased to 64.

## 5 Discussion and conclusions

IPF is a chronic progressive disease of unknown etiology. With approximately 5,000 new cases each year and 5-year survival. These incidence and mortality rates are higher than many other cancers. Furthermore, no therapy has been proven effective in altering the prognosis of IPF. Thus, IPF is a chronic, incurable, and progressively fatal disease [1, 23]. A key mediator in epithelial repair and the pathogenesis of IPF is TGF-*β*. Active TGF-*β* is an essential mediator of the profibrotic effects of mesenchymal cells being able to induce transdifferentiation of resident fibroblasts and fibrocytes into myofibroblasts [8–10, 33]. In this article, the model problems describe the space of lung alveoli using the homogenized diffusion equation. We have proposed a mathematical optimal control problem for the first time for treating IPF. We control the TGF-*β* for myofibroblast diffusion when classical methods are used. Five model problems are presented. In the first model problem, the dynamic system of myofibroblast diffusion is solved using finite difference techniques. For the second model problem, we suggest the optimal control problem for myofibroblast diffusion where there is no constraint on TGF-*β*. It is observed that the TGF-*β* control function is negative in this kind of simulation, which is unrealistic and it is needed to be corrected. The third model problem includes a non-negative constraint for TGF-*β*. In Fig 6(B), it is shown that by this kind of simulation, TGF-*β* stays positive (greater than zero). By controlling TGF-*β*, myofibroblast density tends to zero, which is consistent with medical facts. The value of TGF-*β* in the third model problem is lower than TGF-*β* in the dynamic system, suggesting that medication be used to reduce the TGF-*β*. It is not easy to reduce TGF-*β* by following a function that changes with time passing. For physicians to develop easier guidelines, the fifth model problem allows physicians to use a drug with a fixed amount during treatment to reduce TGF-*β*. According to the fifth model problem, reducing TGF-*β* seems simpler than the third model problem. In the fourth model problem, the dynamic fibroblast system is proposed based on Fig 1. It is solved using the central finite difference techniques. In the fifth model problem, we formulate an optimal control problem in which the fibroblast diffusion equation and myofibroblast diffusion equation are considered dynamic systems. The numerical solution for the fifth model problem yields a constant TGF-*β* under optimal conditions. Moreover, the fifth model problem is applicable for using newly explored like an anti-TGF-*β* Pirfenidone medicine [37]. The numerical results in this paper confirm vanishing myofibroblast by controlling TGF-*β*. These results correspond to the fact given in [38, 39], as they say: myofibroblast proliferation declines, myofibroblast contraction ceases, and apoptosis occurs. Reduced myofibroblast density and apoptosis prevent collagen from forming within the ECM [39].

## Supporting information

S1 File (PDF)
