# Supplementary material for: Optimal control of TGF-β to prevent formation of pulmonary fibrosis
Source: PLoS One. 2022 Dec 30;17(12):e0279449. doi: 10.1371/journal.pone.0279449 (PMC9803315; doi:10.1371/journal.pone.0279449)
Supplement: S1 File — (PDF) [file pone.0279449.s001.pdf]

Figs 4 and 5 code

```
import numpy as np

import matplotlib.pyplot as plt

import scipy

from scipy.sparse import diags

nx=6# GRID POINTS on space interval

ny=6# GRID POINTS on space interval

nf=300 # final time

dx=1/nx

dy=1/ny

D_m = 1.47e-5

########  Time step

#dt1=dx**2/(4*D_m ) or dt1=(dx**2*dy**2)/(2*D_m *(dx**2+dy**2))for
satibility dt<dt1

dt=0.5

#########     Creates grids

x=np.linspace(0,1,nx)

y=np.linspace(0,1,ny)

t=np.arange(0,nf,dt)

nt=t.size  # GRID POINTS on time interval

#########    initial conditions state,control,Ricati

m=np.ones((nx**2,ny**2,nt))*8.5e-3####  state with package

mn=np.ones((nx**2,ny**2,nt))*8.5e-3#### state with package

mwithout=np.ones((nx**2,ny**2,nt))*8.5e-3#### without package

mnwithout=np.ones((nx**2,ny**2,nt))*8.5e-3#### without package
```

```
#######       assign initial conditions
f=4.75e-3
gama=127/343
a=.11
r1=(dt*D_m*nx**2)
r1 = a*r1/gama
rm=r1/dt
r2=(dt*D_m*ny**2)
r2 = a*r2/gama
T_B=2.51e-12
T_B=343*(T_B/127)
K_TB=1e-10
d_m=1.66e-2
landa_mft=.12
landa_mfg=.12
G=.58e-8
G=343*(G/127)
K_G=1.5e-8
alfa=2*rm-d_m
u1=np.ones((nx**2,ny**2,nt))*T_B
u2=np.ones((nx**2,ny**2,nt))*T_B
pp=np.zeros((nx**2,ny**2,nt))##riccti  without package
S=np.ones((nx**2,ny**2,nt))#riccti
SS=np.ones((nx**2,ny**2,nt))#riccti without package
Boundry=np.zeros((nx**2,ny**2,nt))
```

```
C=np.ones((nx**2,ny**2,nt))*landa_mfg*(G/(G+K_G))*f
########      System behavior
def F(m):
  return (landa_mft*(T_B/(T_B+K_TB))+landa_mfg*(G/(G+K_G)))*f-d_m*m
###################################################################################
######## without control
mb=np.ones((nx,ny,nt))*8.5e-3
mbn=np.ones((nx,ny,nt))*8.5e-3


###### run through nt times
for k in range(0,nt-1):
   mb=mbn.copy()

mbn[int(.3/dx):int(2*.3/dx)+1,int(.3/dy):int((2*.3)/dy)+1,k+1]=(mb[int(.3/dx):int(2*.3/dx)+1,int(.3/dy):int((2*.3)/dy)+1,k]
   +r1*(mb[int(.3/dx):int(2*.3/dx)+1,int(.3/dy)+1:int((2*.3)/dy)+2,k]-2*mb[int(.3/dx):int(2*.3/dx)+1,int(.3/dy):int((2*.3)/dy)+1,k]+
   mb[int(.3/dx):int(2*.3/dx)+1,int(.3/dy)-1:int((2*.3)/dy),k])+
   r2*(mb[int(.3/dx)+1:int((2*.3)/dx)+2,int(.3/dy):int((2*.3)/dy)+1,k]-2*mb[int(.3/dx):int(2*.3/dx)+1,int(.3/dy):int((2*.3)/dy)+1,k]+
   mb[int(.3/dx)-1:int((2*.3)/dx),int(.3/dy):int((2*.3)/dy)+1,k]))+dt*F(mb[int(.3/dx):int(2*.3/dx)+1,int(.3/dy):int((2*.3)/dy)+1,k])

mb[int(.3/dx):int(2*.3/dx)+1,int(.3/dy):int((2*.3)/dy)+1,k+1]=mb[int(.3/dx):int(2*.3/dx)+1,int(.3/dy):int((2*.3)/dy)+1,k]+dt*mbn[int(.3/dx):int(2*.3/dx)+1,int(.3/dy):int((2*.3)/dy)+1,k]# update m with oyler
######## boundary
```

```
   mb[int(.3/dx),:,k+1]=mb[int(.3/dx),:,k]+r1*(mb[int(.3/dx)+1,:,k]+mb[-1,:,k])-2*mb[int(.3/dx),:,k]+dt*F(mb[int(.3/dx),:,k])

   mb[:,int(.3/dx),k+1]=mb[:,int(.3/dx),k]+r1*(mb[:,int(.3/dx)+1,k]+mb[:,-1,k])-2*mb[:,int(.3/dx),k]+dt*F(mb[:,int(.3/dx),k])


mb[int(2*.3/dx)+1,:,k+1]=mb[int(2*.3/dx)+1,:,k]+r1*(mb[int(.3/dx),:,k]+mb[int(2*.3/dx),:,k])-2*mb[int(2*.3/dx)+1,:,k]+dt*F(mb[int(2*.3/dx)+1,:,k])


mb[:,int(2*.3/dx)+1,k+1]=mb[:,int(2*.3/dx)+1,k]+r1*(mb[:,int(.3/dy),k]+mb[:,int(2*.3/dx),k])-2*mb[:,int(2*.3/dx)+1,k]+dt*F(mb[:,int(2*.3/dx)+1,k])

#####################################################################

########  with control

#########     matrix A,B,Q,R

A0 = [rm*np.ones(nx-1),alfa*np.ones(nx),rm*np.ones(nx-1)]

offset = [-1,0,1]

A0 = diags(A0,offset).toarray()

I=np.ones((nx,nx))

zero=np.zeros((nx,nx))

A1=np.concatenate((A0,rm*I),axis=0  )

A1=np.concatenate((A1,zero),axis=0  )

A1=np.concatenate((A1,zero),axis=0  )

A1=np.concatenate((A1,zero),axis=0  )

A1=np.concatenate((A1,zero),axis=0  )

##

A2=np.concatenate((rm*I,A0),axis=0  )

A2=np.concatenate((A2,rm*I),axis=0  )

A2=np.concatenate((A2,zero),axis=0  )
```

```
A2=np.concatenate((A2,zero),axis=0  )
A2=np.concatenate((A2,zero),axis=0  )
###
A3=np.concatenate((zero,rm*I),axis=0  )
A3=np.concatenate((A3,A0),axis=0  )
A3=np.concatenate((A3,rm*I),axis=0  )
A3=np.concatenate((A3,zero),axis=0  )
A3=np.concatenate((A3,zero),axis=0  )
###
A4=np.concatenate((zero,zero),axis=0  )
A4=np.concatenate((A4,rm*I),axis=0  )
A4=np.concatenate((A4,A0),axis=0  )
A4=np.concatenate((A4,rm*I),axis=0  )
A4=np.concatenate((A4,zero),axis=0  )
###
A5=np.concatenate((zero,zero),axis=0  )
A5=np.concatenate((A5,zero),axis=0  )
A5=np.concatenate((A5,rm*I),axis=0  )
A5=np.concatenate((A5,A0),axis=0  )
A5=np.concatenate((A5,rm*I),axis=0  )
###
A6=np.concatenate((zero,zero),axis=0  )
A6=np.concatenate((A6,zero),axis=0  )
A6=np.concatenate((A6,zero),axis=0  )
A6=np.concatenate((A6,rm*I),axis=0  )
```

```
A6=np.concatenate((A6,A0),axis=0  )
###
A=np.concatenate((A1,A2),axis=1  )
A=np.concatenate((A,A3),axis=1  )
A=np.concatenate((A,A4),axis=1  )
A=np.concatenate((A,A5),axis=1  )
A=np.concatenate((A,A6),axis=1  )
#D=np.linalg.det(A)
B0=landa_mft*f*np.ones((nx,nx))
B1=np.concatenate((B0,I),axis=0  )
B1=np.concatenate((B1,zero),axis=0  )
B1=np.concatenate((B1,zero),axis=0  )
B1=np.concatenate((B1,zero),axis=0  )
B1=np.concatenate((B1,zero),axis=0  )
##
B2=np.concatenate((I,B0),axis=0  )
B2=np.concatenate((B2,I),axis=0  )
B2=np.concatenate((B2,zero),axis=0  )
B2=np.concatenate((B2,zero),axis=0  )
B2=np.concatenate((B2,zero),axis=0  )
###
B3=np.concatenate((zero,I),axis=0  )
B3=np.concatenate((B3,B0),axis=0  )
B3=np.concatenate((B3,I),axis=0  )
B3=np.concatenate((B3,zero),axis=0  )
```

```
B3=np.concatenate((B3,zero),axis=0  )
###
B4=np.concatenate((zero,zero),axis=0  )
B4=np.concatenate((B4,zero),axis=0  )
B4=np.concatenate((B4,I),axis=0  )
B4=np.concatenate((B4,B0),axis=0  )
B4=np.concatenate((B4,zero),axis=0  )
###
B5=np.concatenate((zero,zero),axis=0  )
B5=np.concatenate((B5,zero),axis=0  )
B5=np.concatenate((B5,I),axis=0  )
B5=np.concatenate((B5,B0),axis=0  )
B5=np.concatenate((B5,I),axis=0  )
###
B6=np.concatenate((zero,zero),axis=0  )
B6=np.concatenate((B6,zero),axis=0  )
B6=np.concatenate((B6,zero),axis=0  )
B6=np.concatenate((B6,I),axis=0  )
B6=np.concatenate((B6,B0),axis=0  )
###
B=np.concatenate((B1,B2),axis=1  )
B=np.concatenate((B,B3),axis=1  )
B=np.concatenate((B,B4),axis=1  )
B=np.concatenate((B,B5),axis=1  )
B=np.concatenate((B,B6),axis=1  )
```

```
#  Q,R
Q1 = [np.zeros(nx**2-1),np.ones(nx**2),np.zeros(nx**2-1)]
offset = [-1,0,1]
Q = diags(Q1,offset).toarray()
# R
R1 = [np.zeros(nx**2-1),np.ones(nx**2),np.zeros(nx**2-1)]
offset = [-1,0,1]
R = diags(R1,offset).toarray()
Boundry[int(.3/dx),:,:]=8.5e-3*rm
Boundry[:,int(.3/dx),:]=8.5e-3*rm
Boundry[int((2*.3)/dy+1),:,:]=mb[int((2*.3)/dy)+1,int((2*.3)/dy)+1,int((2*.3)/dy)+1]*rm
Boundry[:,int((2*.3)/dy+1),:]=mb[int((2*.3)/dy)+1,int((2*.3)/dy)+1,int((2*.3)/dy)+1]*rm
for k in range(nt-1,0,-1):
    pp[:,:,k-1]= pp[:,:,k]+dt*(Q+pp[:,:,k]*A+A.T*pp[:,:,k]-(pp[:,:,k]*B)*R*(B.T*pp[:,:,k]))#R^(-1)=R
    SS[:,:,k-1]=SS[:,:,k]+dt*(A.T-(pp[:,:,k]*B*B.T)*SS[:,:,k]-pp[:,:,k])
######  run through nt times
## e,s for package solve_continuous_are for Ricati eqution
e =[np.ones(nx**2)]
offset = [0]
e =diags(e,offset).toarray()
N=0
s=np.zeros((nx**2,nx**2))
for k in range(0,nt-1):
```

```
   u=u1.copy()
   m=mn.copy()
    ########   with package solve_continuous_are for Ricati eqution
   p=scipy.linalg.solve_continuous_are(A, B, Q, R,e=None, s=None,
balanced=True)
   S[:,:,k+1]=S[:,:,k]-dt*(A.T-(p*B*B.T)*S[:,:,k]+p)
   KK=-R*((B.T)*p)#
   u1[:,:,k+1]=KK*mn[:,:,k]+(Boundry[:,:,k]+rm*C[:,:,k])*B.T*S[:,:,k]
   mn[:,:,k+1]=m[:,:,k]+dt*(A*m[:,:,k]+B*u1[:,:,k]+Boundry[:,:,k]+rm*C[:,:,k])
u_T=u1*K_TB/(1-u1)
###################
###################
for k in range(0,nt-1):
   uu=u2.copy()
   mwithout=mnwithout.copy()
    ########   with out packege
   pp[:,:,k+1]= pp[:,:,k]-dt*(Q+pp[:,:,k]*A+A.T*pp[:,:,k]-
(pp[:,:,k]*B)*R*(B.T*pp[:,:,k]))#R^(-1)=R
   SS[:,:,k+1]=SS[:,:,k]-dt*(A.T-(pp[:,:,k]*B*B.T)*SS[:,:,k]-pp[:,:,k])
   K1=-R*(B.T)*pp[:,:,k]
   u2[:,:,k+1]=K1*mnwithout[:,:,k]+(Boundry[:,:,k]+rm*C[:,:,k])*B.T*SS[:,:,k]

mnwithout[:,:,k+1]=mwithout[:,:,k]+dt*(A*mwithout[:,:,k]+B*u2[:,:,k]+Boundry[:
,:,k]+rm*C[:,:,k])#
uu_T=u2*K_TB/(1-u2)
time=t[1:-1:110]
mstar=mwithout[:,:,1:-1:110]
```

```
ustar=u_T[:,:,1:-1:110]
print("m=",mb[2,2,2])
######################################################################
########  plotting
fig = plt.figure(1)
plt.plot(t,uu_T[12,12,:],linewidth=2,color='r',label='Euler approximation')
plt.plot (time,ustar[12,12,:],'*',linewidth=2,label='Scipy.linalg')
plt.xlabel('time (day)')
plt.ylabel('$T_{GF}$')
plt.xlim(xmin=0)
plt.title('$dt=0.1$, (A)')
plt.grid(True)
plt.legend()
plt.show()
fig = plt.figure(2)
############################################
plt.plot(t,mbn[2,2,:],color='g',linewidth=2, label='First model problem')
plt.plot(t,mn[2,2,:],color='r',linewidth=2,label='Second model problem')
plt.plot(time,mstar[2,2,:],'*',label='Second model problem (scipy.linalg )')
plt.title('$dt=0.1$, (A)')
plt.xlabel('time (day)')
plt.ylabel('Myofibroblast density')
plt.xlim(xmin=0)
plt.ylim(ymin=0)
plt.grid(True)
```

```
plt.legend()
plt.show()
```
